# Antibiotic-associated *Enterococcus* expansion in the gastrointestinal tract precedes infected necrosis in acute necrotizing pancreatitis

**DOI:** 10.1080/19490976.2026.2670039

**Published:** 2026-05-15

**Authors:** Fons F. van den Berg, Hannah S. Pauw, Hester C. Timmerhuis, Marc G. Besselink, Yama Issa, Marco J. Bruno, Pieter Jan de Jonge, Harry van Goor, Erwin J. M. van Geenen, Rutger Quispel, Wim van de Vrie, Adriaan Tan, Muhammed Hadithi, Niels G. Venneman, Rogier P. Voermans, Jeroen M. Jansen, Ben J. Witteman, Matthijs P. Schwartz, Roy L. J. van Wanrooij, Alexander C. Poen, Peter van Duijvendijk, Marie-Paule Anten, Tessa E. H. Römkens, Elske Sieswerda, Merel M. Tielemans, Jeanin E. van Hooft, Marja A. Boermeester, Robert C. Verdonk, Hjalmar C. van Santvoort

**Affiliations:** a Department of Medical Microbiology and Infection Prevention, Amsterdam UMC Location AMC, Amsterdam, The Netherlands; b Amsterdam Institute for Immunology and Infectious Diseases, Amsterdam UMC, Amsterdam, The Netherlands; c Department of Surgery, St Antonius Hospital, Nieuwegein, The Netherlands; d Department of Research and Development, St Antonius Hospital, Nieuwegein, The Netherlands; e Department of Surgery, Amsterdam UMC Location AMC, Amsterdam, The Netherlands; f Amsterdam Gastroenterology Endocrinology Metabolism, Amsterdam UMC, Amsterdam, The Netherlands; g Department of Surgery, Leiden University Medical Center, Leiden, The Netherlands; h Department of Gastroenterology and Hepatology, Erasmus MC, Rotterdam, The Netherlands; i Department of Surgery, Radboud University Medical Center, Nijmegen, The Netherlands; j Department of Gastroenterology and Hepatology, Radboud University Medical Center, Nijmegen, The Netherlands; k Department of Gastroenterology and Hepatology, Reinier de Graaf Hospital, Delft, The Netherlands; l Department of Gastroenterology, Albert Schweitzer Hospital, Dordrecht, The Netherlands; m Department of Gastroenterology, Canisius Wilhelmina Hospital, Nijmegen, The Netherlands; n Department of Gastroenterology and Hepatology, Maasstad Hospital, Rotterdam, The Netherlands; o Department of Gastroenterology and Hepatology, Medisch Spectrum Twente, Enschede, The Netherlands; p Department of Gastroenterology and Hepatology, Amsterdam UMC Location AMC, Amsterdam, The Netherlands; q Department of Gastroenterology, OLVG, Amsterdam, The Netherlands; r Department of Gastroenterology and Hepatology, Gelderse Vallei Hospital, Ede, The Netherlands; s Department of Internal Medicine and Gastroenterology, Meander Medical Center, Amersfoort, The Netherlands; t Department of Gastroenterology and Hepatology, Amsterdam UMC Location VUmc, Amsterdam, The Netherlands; u Department of Gastroenterology and Hepatology, Isala, Zwolle, The Netherlands; v Department of Surgery, Gelre Hospitals, Apeldoorn, The Netherlands; w Department of Gastroenterology, Franciscus Gasthuis & Vlietland, Rotterdam, The Netherlands; x Department of Gastroenterology and Hepatology, Jeroen Bosch Hospital, Hertogenbosch, The Netherlands; y European Clinical Research Alliance on Infectious Diseases (ECRAID), Utrecht, The Netherlands; z Julius Center for Health Sciences and Primary Care, Utrecht University, Utrecht, The Netherlands; aa Department of Gastroenterology and Hepatology, Bravis Hospital, Roosendaal, The Netherlands; ab Department of Laboratory Medicine, Radboud University Medical Center, Nijmegen, The Netherlands; ac Department of Gastroenterology and Hepatology, Leiden University Medical Center, Leiden, The Netherlands; ad Department of Gastroenterology and Hepatology, St Antonius Hospital, Nieuwegein, The Netherlands; ae Department of Surgery, UMC Utrecht, Utrecht, The Netherlands

**Keywords:** Intestinal colonization, necrotizing pancreatitis, intestinal microbiota

## Abstract

Infected pancreatic necrosis complicates 30% of necrotizing pancreatitis cases, with intestinal bacterial translocation as the presumed mechanism. To characterize gastrointestinal microbiota dynamics preceding infection, we performed a predefined subgroup analysis of the POEMA cohort, a prospective multicenter microbiota study across 20 Dutch hospitals. Of 276 patients with acute pancreatitis, 57 with necrotizing pancreatitis underwent twice-weekly rectal and salivary sampling for 30 d, analyzed using 16S rRNA sequencing. Twenty (35%) developed infected necrosis. After adjustment for baseline disease severity, biliary etiology, and prior antibiotic exposure, gastrointestinal *Enterococcus* colonization was associated with subsequent infected necrosis (rectal HR 4.48, 95% CI 1.51–13.28; salivary HR 5.37, 95% CI 1.72–16.79), typically preceding clinical diagnosis by 3 weeks; results were similar when adjusting for early extra-pancreatic infection instead of disease severity. *Enterococcus* colonization developed during admission, predominantly in the first 2 weeks, rather than being present at baseline. Early antibiotic use—without documented infection in 40%—was associated with *Enterococcus* colonisation (HR 4.99, 95% CI 1.57–15.80), reduced butyrate-producer abundance, and infected necrosis (HR 3.56, 95% CI 1.23–10.28). Whether this relationship is causal warrants further investigation, but if confirmed, it would warrant a more restrictive antibiotic policy in early acute pancreatitis.

## Introduction

Infected (peri-)pancreatic necrosis (IPN) is a severe complication that occurs in approximately 30% of patients with acute necrotizing pancreatitis.^1^ IPN is associated with mortality rates of 15%–30% and necessitates treatment that often includes multiple percutaneous or endoscopic procedures and prolonged, broad-spectrum antibiotic administration.^1^ Intestinal pathogens that normally reside in low abundance in the intestinal microbiota of healthy individuals, such as *Enterobacterales* and enterococci, are frequently found in cultures of IPN.^2^ Bacterial seeding of (peri-)pancreatic necrosis following systemic dissemination of intestinal pathogens is the presumed cause. This is facilitated by an increase in intestinal permeability caused by severe local and systemic inflammation. Systemic immunosuppression, as a result of initial severe systemic inflammation, predisposes patients to infected necrosis, which typically occurs 2–4 weeks after hospital admission.^3-5^ This enables invading pathogens to disseminate to necrotic tissue, causing infected necrosis.^6^^,^^7^

Currently, clinicians cannot reliably identify which patients will develop IPN, and diagnosis relies on clinical deterioration (fever, sepsis), radiological signs of infection (gas in necrotic collections), or positive cultures from fine-needle aspiration or drainage procedures. No validated biomarkers exist to stratify patients with necrotizing pancreatitis for trials of targeted preventive interventions, such as antibiotic prophylaxis, probiotics, or enteral nutrition, which may succeed in high-risk subgroups where previous unstratified approaches have failed. Understanding the dynamics of gastrointestinal colonization preceding infection may reveal modifiable risk factors, given the established gut-driven hypothesis. Previous cross-sectional studies using single early time-points were inadequately designed to identify microbiota perturbations leading up to infected necrosis, given the multiple-week interval between hospital admission and diagnosis of infected necrosis.^8-14^

To address this gap, this prospective multicenter observational study explores the hypothesis that early intestinal colonization with pathogenic bacteria drives progression to IPN in patients with necrotizing pancreatitis. The aim was to identify microbiota-based biomarkers through longitudinal sampling of the upper and lower gastrointestinal tract during the first 30 d of admission.

## Materials and methods

### Ethics approval

The study was approved by the Medical Research Ethics Committees United (MEC-U) with registration number NL67406.100.18. All included patients provided written informed consent for participation, and the study was conducted in accordance with the principles of the Declaration of Helsinki.

### Study design, patient population

This was a study in a predefined subgroup of patients with necrotizing pancreatitis, that were included as part of a prospective, observational patient cohort (POEMA cohort). The POEMA cohort consists of a predefined sample size of patients with first-episode acute pancreatitis who presented at 20 participating Dutch hospitals between October 2019 and January 2022. Characteristics of the complete POEMA patient were previously published.^15^ Acute pancreatitis was defined as having at least two of the following: (a) abdominal pain consistent with acute pancreatitis, (b) serum lipase or amylase levels at least three times above the upper limit of normal, and (c) characteristic findings of acute pancreatitis on abdominal computed tomography (CT), magnetic resonance imaging, or ultrasonography. Necrotizing pancreatitis was defined as having signs of (peri)pancreatic necrosis on contrast-enhanced computed tomography (CT) imaging. Patients who met the following criteria were excluded from participation: age younger than 18 y, pregnancy, disease onset more than 72 hours ago, a history of (recurrent) acute or chronic pancreatitis, antibiotic use within the last 2 months. The trial was registered (NL-OMON29684) and the study protocol has been published as part of a thesis.^16^ This study was reported in accordance with the Strengthening the Reporting of Observational Studies in Epidemiology (STROBE) guidelines.^17^

### Clinical data

A patient questionnaire, collected at inclusion, included self-reported medical and lifestyle information, such as alcohol use, smoking, antibiotic treatment within 6 months prior to admission and proton pump inhibitor use, as described before.^15^ Clinical data, including antibiotic administration and microbiology results, were prospectively collected from the electronic medical records and entered in an Electronic Data Capture database (Castor EDC). Dutch Pancreatitis Study Group (DPSG) investigators at the participating centers contributed to local treatment protocols based on consensus within the DPSG and current international guidelines, including a step-up approach to drainage and avoiding unwarranted (prophylactic) antibiotics. Antibiotic treatment decisions were at the discretion of the treating physician, guided by institutional protocols based on Dutch national guidelines (SWAB). The follow-up was at 90 d following the initial admission. Predicted severity of pancreatitis was determined within 48 h of hospital admission, using the following criteria: APACHE-II ≥ 7, Imrie ≥ 3, and/or C-reactive protein (CRP) > 150. The revised Atlanta classification was used for severity stratification.^18^

The primary outcome was infected necrosis, defined as: (1) the presence of air configurations in a (peri)pancreatic collection, (2) bacterial or fungal growth of a fine-needle aspiration (FNA) of a (peri)pancreatic collection or a drainage culture according to the local microbiology report, or (3) a percutaneous or endoscopic drainage procedure that was performed because of the suspicion of infected necrosis without available cultures. Necrosis cultures from FNA, EUS-guided catheter-drainage, or necrosectomy, percutaneous catheter drainage, video-assisted retroperitoneal drainage or necrosectomy were considered for determining the causative pathogen, but only when samples were collected within 24 h of drain placement to avoid mislabeling drain-colonizing microbes as causative pathogens.

### Sample collection

Rectal swabs were used as a representative sample of the lower gastrointestinal tract because it provides comparable profiles as stool,^19-21^ and the difficulty of consistent and timely collection of stool in this clinically ill patient population. A rectal swab and saliva sample were collected by hospital staff as soon as possible following enrollment, using commercial kits (respectively, Omnigene OM-501 and P-117) according to the product instructions. These kits are designed to stabilize samples for respectively 30 d and a year at room temperature. Paired rectal swab and saliva samples were collected twice weekly in patients with a predicted severe disease course. Sample collection was stopped in case of hospital discharge, death, occurrence of infected necrosis, or at the maximum of eight sample collections. Samples were sent by mail at ambient temperature to a central laboratory and transferred to −80 °C within 72 h from collection until further processing.

### DNA extraction and 16S rRNA gene sequencing

DNA was extracted from rectal swabs and saliva, 16S rRNA gene amplicons were generated and sequenced using an Illumina MiSeq platform (detailed methods in Extended Methods). Bioinformatic processing was performed using USEARCH^22^ with taxonomy assigned using the RDP classifier^23^ and SILVA 16S ribosomal database V132.^24^ Quality filtering removed samples with <10,000 sequences and ASVs with relative abundance <0.1% per sample (detailed bioinformatic pipeline in Extended Methods).

### Statistical analyses

Predefined analyses comparing patients with sterile and infected necrotizing pancreatitis included alpha diversity, quantified using the inverse Simpson diversity index. Beta diversity was assessed using principal coordinates analysis on Bray–Curtis distances. Longitudinal changes in diversity metrics were modeled using linear mixed-effects models with infection status, time, and their interaction as fixed effects, and subject as a random intercept. Differential abundance testing on genus-level aggregated data was performed using Ancom-BC2^25^ and MaAsLin2^26^ with FDR-adjusted *p* values (*q* values) <0.05 considered significant. Given that untargeted differential abundance methods have limited power in small cohorts and may miss clinically relevant signals, we additionally performed hypothesis-driven analyses focusing on bacterial genera known to cause IPN based on a previous culture study from our group.^2^ These targeted longitudinal taxa analysis used zero-inflated negative binomial mixed-effects models to handle excess zeros and Cox proportional hazards models that investigated clinical and microbiome predictors of infected necrosis and colonization. Site-specific colonization thresholds were defined based on a biological rationale: for rectal samples, where *Enterococcus* is commonly detected at low abundance, a threshold of >5% relative abundance was used to indicate overgrowth, with sensitivity analyses evaluating thresholds from 1% to 10% and log10-transformed continuous abundance; for salivary samples, where *Enterococcus* is rarely detected in health, any detection was considered meaningful. Microbiome parameters were analyzed both as baseline and as time-dependent covariates, and included the Inverse Simpson Index (alpha-diversity) and taxa that corresponded with bacteria frequently found in infected necrosis cultures. The multivariate models were adjusted for biliary etiology, prior antibiotic use, and baseline severity (highest APACHE-II score within 48 h of admission). A secondary model with early extra-pancreatic infection as confounder instead of APACHE-II was used to further evaluate confounding by indication. These variables were analyzed separately because early infection may mediate the effect of baseline severity on IPN; simultaneous adjustment could introduce collider bias. Discriminative ability of *Enterococcus* abundance and C-reactive protein for infected necrosis prediction was assessed using time-dependent landmark analyses. Missing values were imputed using multiple imputation by chained equations (MICE). Statistical significance was defined as *p* < 0.05. Detailed statistical methods, including software packages and model specifications, are provided in Extended Methods.

### Role of the funding source

The funding sources had no role in study design; in the collection, analysis, and interpretation of data; in the writing of the report; and in the decision to submit the paper for publication.

## Results

### Description of study population and samples

A total of 276 patients with acute pancreatitis were included in the POEMA study. Characteristics of this cohort are published elsewere.^15^ The predefined subgroup of this study included 57 patients with necrotizing pancreatitis (17.9%), of whom 20 (35%) developed infected necrosis. Infected necrosis was diagnosed by microbiological and/or radiological criteria in 19, and one by drainage on clinical suspicion without microbiological confirmation. Baseline characteristics and clinical outcomes of these patients are presented in Table 1. Biliary and alcoholic etiology were most frequent (respectively 50.9% and 21.8%). A total of 321 samples were collected and sequenced with 16S rDNA amplicon sequencing. In total, 154 rectal swabs and 152 saliva samples had a sufficient depth of >10,000 reads (Supplementary Figure S1). This resulted in respectively 6,860,955 (median 41,840, range 20,331–89,858) and 6,886,959 (median 39,184, range 26,578–88,573) sequences (Supplementary Figure S2).

**Table 1.** Characteristics and clinical outcomes of 57 patients with necrotizing pancreatitis.

	Sterile necrosis *n* = 37	Infected necrosis *n* = 20	Total *n* = 57
**Patient characteristics**			
Age, y, median (IQR)	59 (47.5–69)	64 (54.2–71.2)	61 (50–70.5)
Sex, male, *n* (%)	22 (62.9%)	12 (60%)	34 (61.8%)
BMI, kg/m², mean (SD)	28.5 (5.4)	29.8 (6)	28.9 (5.6)
ASA 1–2, *n* (%)	32 (91.4%)	18 (90%)	50 (90.9%)
ASA 3–4, *n* (%)	3 (8.6%)	2 (10%)	5 (9.1%)
Smoking, *n* (%)	7 (20%)	4 (20%)	11 (20%)
Daily alcohol use, *n* (%)	13 (37.1%)	11 (55%)	24 (43.6%)
PPI use, *n* (%)	4 (11.4%)	6 (30%)	10 (18.2%)
Stool type constipation (BSS 1–2), *n* (%)	9 (25.7%)	2 (10%)	11 (20%)
Antibiotics before admission, *n* (%)	11 (31.4%)	2 (10%)	13 (23.6%)
**Risk prediction (within 48 h of admission)**			
CRP, mg/L, median (IQR)	125 (33–225.5)	167.5 (44.2–219.2)	139 (36–222.5)
Imrie score, median (IQR)	2 (1–2)	2 (1–3)	2 (1–3)
APACHE-II score, median (IQR)	9 (7–12.5)	11.5 (8–16.5)	10 (7.5–13)
Predicted severe AP*, *n* (%)	29 (82.9%)	19 (95%)	48 (87.3%)
**Disease etiology**			
Biliary, *n* (%)	20 (57.1%)	8 (40%)	28 (50.9%)
Alcoholic, *n* (%)	6 (17.1%)	6 (30%)	12 (21.8%)
Idiopathic, *n* (%)	3 (8.6%)	2 (10%)	5 (9.1%)
Other, *n* (%)	6 (17.1%)	4 (20%)	10 (18.2%)
**Clinical outcome**			
Time of diagnosis necrotizing pancreatitis, median days (range)	5 (0–63)	6 (2–53)	6 (0–63)
Time of diagnosis infected necrosis, median days (range)	NA	38 (4–159)	38 (4–159)
Extra-pancreatic infection, *n* (%)	12 (34.3%)	14 (70%)	26 (47.3%)
Pancreatic intervention procedures, *n* (%)	1 (2.9%)	16 (80%)	17 (30.9%)
Death, *n* (%)	1 (2.9%)	1 (5%)	2 (3.6%)

IQR = interquartile range, BMI = body mass index, ASA = American Society of Anesthesiologists classification, PPI = proton pump inhibitor, BSS = Bristol Stool Scale, CRP = C-reactive protein, APACHE-II = Acute Physiology and Chronic Health Evaluation II, AP = acute pancreatitis, CTSI = CT severity index, IPN = infected pancreatic necrosis, SD = standard deviation.

^*^ CRP > 150, Imrie > 2, and/or APACHE > 7.

### Infection status of necrosis is associated with altered microbiota diversity

**Figure 1.**
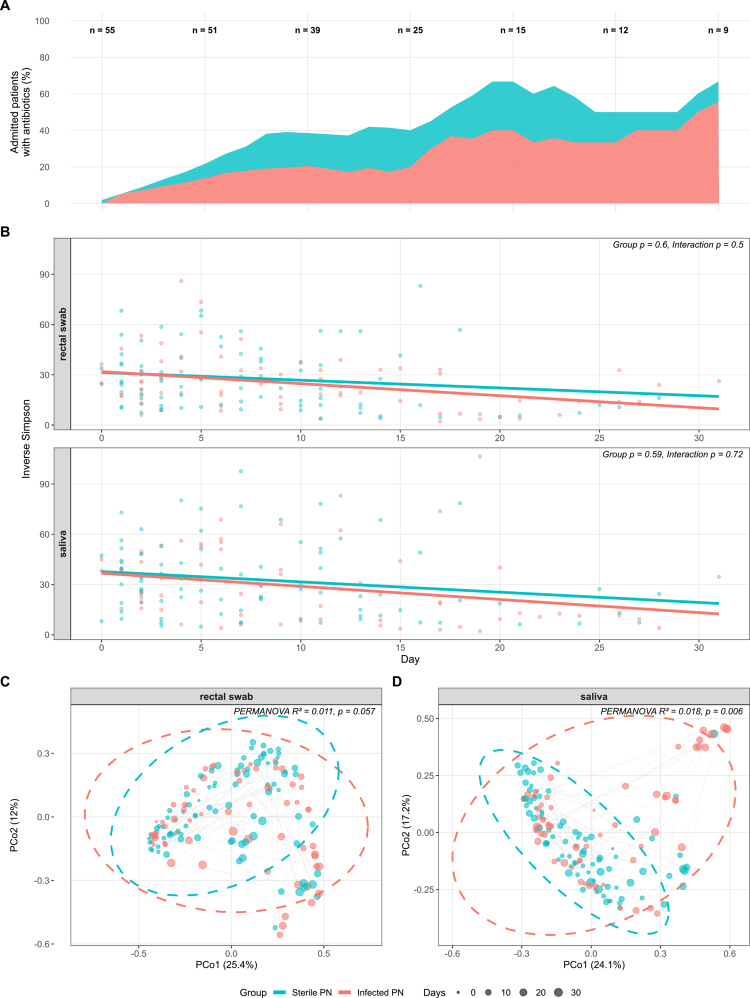
Alpha- and beta-diversity in patients with sterile versus infected necrotizing pancreatitis. (A) Proportion of patients with sterile (blue) or infected (red) necrotizing pancreatitis that are administered antibiotics during admission. The total number of admitted patients is displayed at the top. (B). Inverse Simpson index as measure of alpha diversity in rectal swabs and saliva. Thick lines represent linear mixed-effects model predictions. *p* values for group effect and group × time interaction are displayed per sample type. (C and D) Principal coordinates analysis (PCoA) on Bray–Curtis distances as measure of beta-diversity in rectal swabs (C) and saliva (D). Dashed ellipses indicate 95% confidence intervals per group. Gray lines connect longitudinal samples within individual patients. Point size reflects sampling day. PERMANOVA *R*² and *p* values are displayed per panel.

Alpha (within-sample) diversity was assessed using the inverse Simpson diversity index and analyzed with linear mixed-effects models using random intercepts to account for within-participant correlation. Both saliva and rectal samples demonstrated significant temporal declines in inverse Simpson diversity (rectal swab: *χ*² = 9.84, *df* = 1, *p* = 0.002; saliva: *χ*² = 8.65, *df* = 1, *p* = 0.003), independent of infection status (Figure 1). This coincides with a steadily increase of antibiotic administration. No significant main effects of infection status (rectal swab: *p* = 0.603; saliva: *p* = 0.593) or group-by-time interactions (rectal swab: *p* = 0.496; saliva: *p* = 0.723) were observed for either sample type (Supplementary Table S1). Model coefficients are provided in Supplementary Table S2.

For beta (between-sample) diversity, principal coordinates analysis was performed on Bray–Curtis distances, followed by linear mixed-effects models fitted to PCoA coordinate values. The first four PCoA axes captured 50.9% (rectal swab) and 59.5% (saliva) of total variance (Supplementary Table S3). Linear mixed-effects models revealed significant temporal changes, with rectal swabs showing time effects on PCo1 (*β* = 0.017, *p* < 0.001), PCo2 (*β* = −0.012, *p* < 0.001), and PCo3 (*β* = 0.006, *p* = 0.011), while saliva demonstrated time effects on PCo1 (*β* = 0.018, *p* < 0.001) and PCo2 (*β* = 0.009, *p* = 0.002). No significant baseline group effects were observed (all *p* > 0.05). However, saliva samples showed significant group-by-time interactions on PCo2 (*β* = −0.009, *p* = 0.032) and PCo3 (*β* = 0.008, *p* = 0.011), indicating differential temporal trajectories between infected and non-infected patients, whereas no significant interactions were observed in rectal swabs (Figure 1C).

### Association of microbial taxa with infected necrosis

Differential abundance analysis using ANCOM-BC2 and MaAsLin2 did not show differentially abundant taxa between groups, neither at baseline nor longitudinally. Hypothesis-driven analyses focused on the most frequently cultured bacteria from infected necrosis cultures obtained during drainage procedure or necrosectomy during a recent Dutch study from our group: *Enterobacterales* (52.4%), enterococci (46.9%), staphylococci (21.8%), and streptococci (17%) (Supplementary Table S4).^2^ Longitudinal relative abundance of these taxa is shown in Supplementary Figure S3. No apparent differences in abundance trajectory were seen between IN and IPN patients, except for *Enterococcus* in rectal swabs and *Staphylococcus* in saliva. At baseline (first obtained sample), there were no significant differences in either of these taxa. Zero-inflated mixed-effects models revealed that decreased probability of excess zeros for *Enterococcus* in saliva samples was significantly associated with infected necrosis (estimate = −7.471, *SE* = 1.262, *p* < 0.001), indicating increased detection of this taxon in participants with infection (Supplementary Tables S5 and S6). The other taxa (*Staphylococcus*, *Streptococcus*, and *Enterobacterales*) demonstrated either no significant associations, or patterns suggesting decreased abundance or increased zero-inflation probability with infection. The average, family-level composition during admission is shown in Supplementary Figure S4. It confirms the outgrowth during admission of *Enterococcaceae* (rectal and saliva) during admission. Both rectal and salivary *Enterococcus* relative abundance is negatively correlated with the Inverse Simpsons index (respectively, *R* = −0.37, *p* < 0.001 and *R* = −0.27, *p* < 0.001; Supplementary Figure S5).

In a subset of patients with IPN and available necrosis cultures, gastrointestinal colonization with the same genus preceded pancreatic isolation (Supplementary Figure S6), consistent with the translocation hypothesis.

### *Enterococcus* as marker of infected necrosis

**Figure 2.**
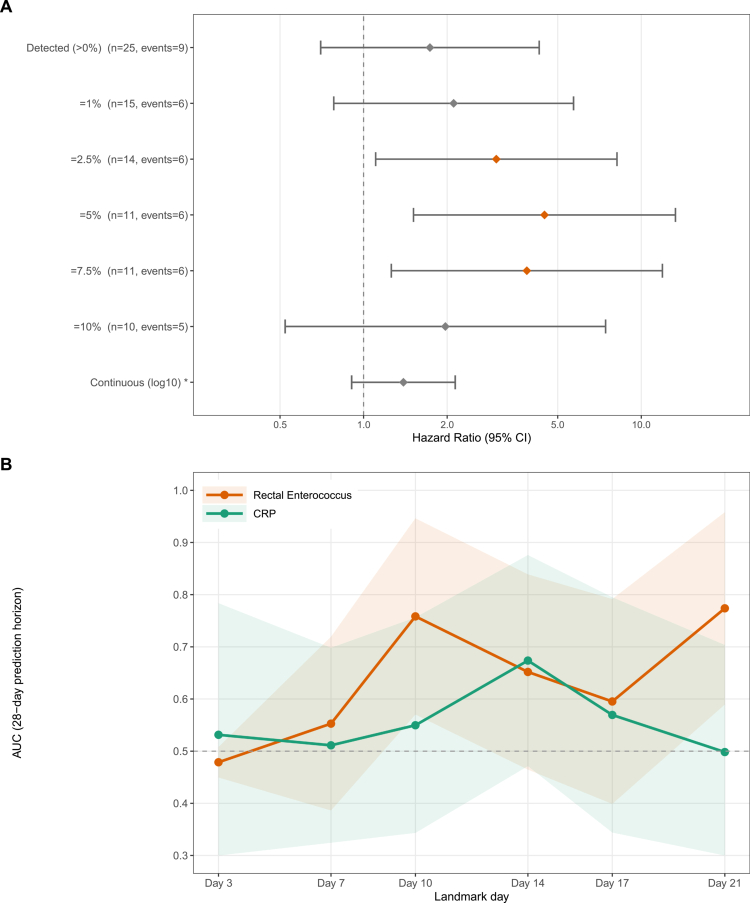
Sensitivity analysis of the association between rectal *Enterococcus* and infected pancreatic necrosis. (A) Hazard ratios from time-dependent Cox models across *Enterococcus* dichotomization thresholds, adjusted for biliary etiology, prior antibiotic use, and APACHE-II score. Orange diamonds indicate *p* < 0.05. *Hazard ratio per log₁₀ increase. (B) Time-dependent AUC comparing rectal *Enterococcus* abundance and CRP for discriminating patients who developed infected necrosis within 28 d from successive landmark days. Marker values reflect last observation carried forward. Shaded areas indicate 95% confidence intervals; dashed line indicates AUC = 0.5.

Cox proportional-hazards modeling was used to determine clinically relevant factors associated with infection development. Patient- and disease-related characteristics that likely impact the microbiota composition, including antibiotic administration, were not associated with infected necrosis (Table 2). Cox proportional hazards regression analysis with time-dependent covariates was performed to examine the association between gut microbiome parameters and the risk of developing infected necrosis over time (Table 3). *Enterococcus* abundances had high proportions of zero values in saliva and was analyzed as detection (at least 1 sequence), or colonization in rectal samples (relative abundance > 5%). After adjusting for biliary etiology, prior antibiotic use, and baseline severity (APACHE-II score), rectal *Enterococcus* colonization was associated with IPN (HR 4.48, 95% CI 1.51–13.28, *p* = 0.007), as was salivary *Enterococcus* detection (HR 5.37, 95% CI 1.72–16.79, *p* = 0.004). A combination of rectal colonization or salivary detection did not provide additional discrimination (HR 4.61, 95% CI 1.53–13.85, *p* = 0.007). The inverse Simpson diversity index was not associated with IPN. Associations were similar when adjusting for early extra-pancreatic infection instead of APACHE-II score (rectal colonization HR 4.26, 95% CI 1.37–13.26; salivary detection HR 6.69, 95% CI 2.19–20.42; Supplementary Table S7). The association was consistent across a range of *Enterococcus* abundance thresholds (Figure 2A). For rectal samples, hazard ratios increased progressively from any detection through ≥5% relative abundance, with significant associations at thresholds between ≥2.5% and ≥7.5%. For salivary samples, any detection showed the strongest association (Supplementary Table S7). Log10-transformed continuous abundance supported a dose–response relationship for both sites, reaching significance for saliva (HR 1.80, *p* = 0.035). Results were similar in the secondary model (Supplementary Table S7).

**Table 2.** Clinical factors associated with infected necrosis.

Predictor	HR (95% CI)	*p* value
*Patient-related*		
Age	1.02 (0.99–1.05)	0.270
Sex	1.18 (0.48–2.89)	0.715
BMI > 30	1.21 (0.50–2.97)	0.671
Daily alcohol intake	1.64 (0.65–4.10)	0.294
Obstipation (BSS 1–2)	0.93 (0.27–3.19)	0.914
PPI use	2.45 (0.94–6.41)	0.067
Prior antibiotics use*	0.35 (0.08–1.52)	0.161
*Disease-related*		
APACHE-II score	1.14 (1.03–1.26)	0.01
Early infection (first week of admission	2.72 (0.99–7.50)	0.053
Etiology (biliary versus other)	0.53 (0.21–1.29)	0.160
Bacteremia prior to IPN diagnosis	0.73 (0.26–2.02)	0.540
Current antibiotic administration^#^	1.97 (0.79–4.90)	0.144

^*^ 2–6 months before admission.

^#^ Time-dependent predictor: antibiotic administration for at least two consecutive days in the week before sample collection.

HR = hazard ratio, CI = confidence interval, BSS = Bristol Stool Scale, BMI = body mass index, PPI = proton pump inhibitor, APACHE-II = Acute Physiology And Chronic Health Evaluation II, IPN = infected (peri)pancreatic necrosis.

**Table 3.** Microbiota associations with infected necrosis.

	Unadjusted		Adjusted	
Predictor	HR (95% CI)	*p* value	HR (95% CI)	*p* value
*Rectal microbiota*				
Inverse Simpson index^#^	0.98 (0.96–1.01)	0.219	0.99 (0.96–1.01)	0.389
* Enterococcus* detection	1.81 (0.74–4.43)	0.195	1.73 (0.70–4.29)	0.234
* Enterococcus* colonization^#^^*^	4.47 (1.68–11.91)	0.003	4.48 (1.51–13.28)	0.007
*Saliva microbiota*				
Inverse Simpson index^#^	0.98 (0.96–1.00)	0.112	0.99 (0.96–1.01)	0.389
* Enterococcus* detection^#^	4.56 (1.64–12.69)	0.004	5.37 (1.72–16.79)	0.004
* Enterococcus* colonization^#^^*^	3.52 (0.80–15.53)	0.097	3.51 (0.71–17.43)	0.125
*Rectal and saliva*				
Rectal *Enterococcus* colonization^#^ and/or salivary *Enterococcus* detection*	3.53 (1.39–8.96)	0.008	4.61 (1.53–13.85)	0.007

HR = hazard ratio, CI = confidence interval.

^*^ Cut-off 5% relative abundance.

^†^ Cut-off 30% relative abundance.

^#^ Time-dependent predictor.

Adjusted for biliary etiology, antibiotic use 2–6 months before admission and highest APACHE-II score within 48 h of admission.

Exploratory time-dependent landmark analyses compared the discriminative ability of rectal *Enterococcus* with C-reactive protein for IPN prediction. At early landmarks (days 3 and 7), neither marker discriminated well (AUC < 0.56). From day 10 onward, *Enterococcus* showed (nonsignificantly) higher point estimates than CRP, with AUCs of 0.758 versus 0.550 at day 10 and 0.774 versus 0.498 at day 21 (Figure 2B, Supplementary Figure S7).

Supplementary Table S8 shows characteristics, microbiome, and microbiology results of the patients with infected necrosis. Rectal *Enterococcus* colonization and/or salivary *Enterococcus* detection was first present at a median of 23 d before the clinical diagnosis of infected necrosis, usually (82%) in the first 2 weeks of hospital admission.

### Early antibiotic administration is a risk factor for *Enterococcus* colonization and infected necrosis

We subsequently examined patient- and disease-related factors associated with rectal *Enterococcus* colonization. Advanced age and female sex were significantly associated with rectal *Enterococcus* colonization (HR 1.06, 95% CI 1.01–1.12, *p* = 0.014; and HR 3.65, 95% CI 1.10–12.15, *p* = 0.034, respectively) (Table 4). Notably, antibiotic administration prior to (2–6 months before admission) and during (defined as antibiotic administration for at least two consecutive days in the week before sample collection) hospital admission was not associated with *Enterococcus* colonization. Antibiotic use during the first week of hospitalization, however, demonstrated a significant association (HR 4.99, 95% CI 1.57–15.80, *p* = −0.006).

**Table 4.** Factors associated with *Enterococcus* colonization.

Predictor	HR (95% CI)	*p* value
*Patient-related*		
Age	1.06 (1.01–1.12)	0.014
Sex	3.65 (1.10–12.15)	0.034
BMI > 30	0.57 (0.15–2.10)	0.395
Daily alcohol intake	0.51 (0.11–2.32)	0.383
Obstipation (BSS 1–2)	1.10 (0.24–5.03)	0.902
PPI use	0.96 (0.21–4.40)	0.963
Prior antibiotic use*	0.31 (0.04–2.38)	0.259
*Disease-related*		
Etiology (biliary versus other)	0.92 (0.30–2.85)	0.884
Current antibiotic administration^#^	2.20 (0.57–8.53)	0.256
Antibiotic administration during first week of admission	4.99 (1.57–15.80)	0.006

HR = hazard ratio, CI = confidence interval, BSS = Bristol Stool Scale, BMI = body mass index, PPI = proton pump inhibitor.

^*^ 2–6 months before admission.

^#^ Time-dependent predictor: antibiotic administration for at least two consecutive days in the week before sample collection.

The proportion of patients that received early antibiotics was higher in patients who later developed infected necrosis (9/20 [45%] versus 6/37 [16.7%]), predominantly second- and third-generation cephalosporins (Supplementary Table S9). Notably, in only 66.6% and 55% of patients with respectively sterile and infected necrosis, early antibiotics were administered for a (suspected) infection, such as bacteremia, pneumonia, or cholangitis. So, 40% of early antibiotic courses lacked documented infection. Second- and third-generation cephalosporines were most frequently administered during the first week; in 13 out of 15 antibiotic-treated patients (86.7%).

**Figure 3.**
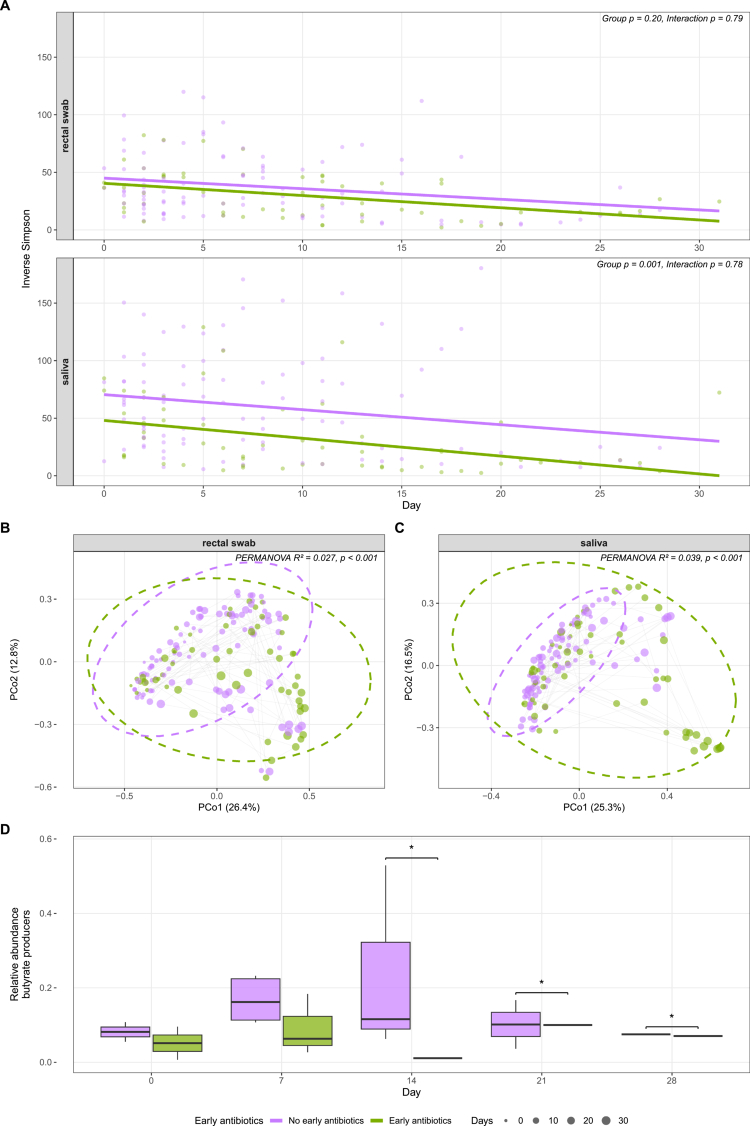
Microbiota dynamics in patients with and without early antibiotic administration. (A) Inverse Simpson index as measure of alpha diversity in rectal swabs and saliva. Thick lines represent linear mixed-effects model predictions. *p* values for group effect and group × time interaction are displayed per sample type. (B and C) Principal coordinates analysis (PCoA) on Bray–Curtis distances as measure of beta-diversity in rectal swabs (B) and saliva (C). Dashed ellipses indicate 95% confidence intervals per group. Gray lines connect longitudinal samples within individual patients. Point size reflects sampling day. PERMANOVA *R*² and *p* values are displayed per panel. (D) Relative abundance of a panel of butyrate-producing genera at different timepoints. *p < 0.05.

Alpha and beta diversity were analyzed in all patients, comparing those who received early antibiotics (*n* = 15) versus no early antibiotics (*n* = 40) using linear mixed-effects models, as described earlier. Saliva samples showed significantly lower baseline diversity in the early antibiotics group that persisted throughout the study period (*χ*² = 10.42, *p* = 0.001; Supplementary Table S10), with the early antibiotics group maintaining 21.9 units lower diversity compared to the no antibiotics group (*p* = 0.044; Supplementary Table S11, Figure 3). No significant interactions between group and time were found in either rectal swab or saliva samples, indicating parallel trajectories between groups. In the saliva microbiome, significant interactions between antibiotics and time were observed across all four PCoA axes (all *p* < 0.01), with positive coefficients for PCo1 (*β* = 0.014, *p* = 0.001) and PCo4 (*β* = 0.008, *p* = 0.004), and negative coefficients for PCo2 (*β* = −0.012, *p* = 0.003) and PCo3 (*β* = −0.009, *p* = 0.004; Supplementary Table S12). Abundance of colonic butyrate-producing bacteria is a measure of intestinal health and low abundance is a risk factor for hospitalization for severe infections.^27^ The relative abundance of butyrate-producing bacteria in rectal swabs was analyzed using a longitudinal linear mixed-effects model. There was a (nonsignificant) difference between the early antibiotics and no antibiotics groups overall (*χ*² = 3.73, *df* = 1, *p* = 0.053, Supplementary Table S13). Butyrate-producer levels did not change significantly over time, and the two groups did not show different trajectories. Post-hoc timepoint comparisons revealed significantly lower butyrate-producer abundance in the early antibiotics group at days 14 (mean difference = 0.050, *p* = 0.025), 21 (mean difference = 0.072, *p* = 0.030), and 28 (mean difference = 0.094, *p* = 0.043), but not at baseline (Figure 3). Model coefficients is provided in Supplementary Table S14. Finally, cox proportional-hazards analysis showed association between early antibiotics and the occurrence of infected necrosis in both the unadjusted (HR 2.90, 95% CI 1.20–7.04, *p* = 0.018) and adjusted model (HR 3.56, 95% CI 1.23–10.28, *p* = 0.019).

## Discussion

In this prospective multicenter study, a predefined subgroup analysis of patients with acute necrotizing pancreatitis showed that early antibiotic administration was associated with gastrointestinal *Enterococcus* expansion, reduced butyrate-producer abundance, and subsequent infected necrosis. Notably, a documented indication for early antibiotic use was lacking in 40% of cases. *Enterococcus* colonization typically developed during the first 2 weeks of hospitalization rather than being present at baseline, coinciding with peak antibiotic exposure. Colonization preceded clinical diagnosis of infected necrosis by a median of 3 weeks, although current step-up protocols defer drainage, meaning definitive diagnosis may lag behind infection onset and this lead time may be overestimated. This temporal pattern would have been missed by single-timepoint assessment. The association between *Enterococcus* colonization and infected necrosis remained after adjustment for baseline severity and was similar when adjusting for early extra-pancreatic infection, arguing against confounding by indication as the sole explanation for these results. While our cohort of 57 patients with necrotizing pancreatitis and 20 infection events limits statistical precision, the effect sizes observed (HR 4–6) are clinically meaningful and consistent across colonization thresholds. In exploratory time-dependent landmark analyses, *Enterococcus* showed modest but increasing discriminative ability from day 10 onward, with higher point estimates than CRP, the most widely used sequential marker in this population. Previous studies have identified early markers of infected necrosis, including lymphocyte count trajectories and rising blood urea nitrogen.^3^^,^^28^ However, these parameters are not modifiable and merely reflect disease severity, in contrast with the microbiota, which may be utilized as targets for both prediction and intervention.

Enterococci are the most frequently cultured Gram-positive bacteria from pancreatic tissue.^2^^,^^29^^,^^30^ While traditionally viewed as commensal organisms with limited virulence in healthy individuals, their pathogenicity may alter under conditions of critical illness and microbiota disruption. For example, *Enterococcus faecalis* dynamically alters virulence gene expression in response to host environmental cues,^31^ and under conditions of antibiotic-induced pertubation, reaches threshold colonization levels that trigger translocation and dissemination.^32^ This transformation from commensals to pathogens-influenced by both the host's compromised state and loss of colonization resistance—may explain their association with poor prognosis in necrotizing pancreatitis.^2^^,^^33^

Consistent with previous cross-sectional microbiome studies, we found no baseline differences in *Enterococcus* abundance between patients who did or did not develop infected necrosis. One study found *Enterococcus* enrichment in neutrophil-associated microbiomes, but not in whole blood samples, of infected acute pancreatitis patients, limiting its practicality as a biomarker.^34^

Although we demonstrated an association between Enterococcus colonization and infected necrosis, this was not the case for half of the patients. Translocation might predominantly occur in the mucosa of the duodenum and small intestine, so the spatioregional distribution of the intestinal microbiota might be explanatory. We applied site-specific colonization thresholds to account for the naturally low *Enterococcus* abundance in saliva versus its established presence in the rectal microbiota, using any detection for oral samples and >5% relative abundance for rectal samples. Sensitivity analyses using continuous abundance and other thresholds pointed in the same direction, indicating that findings were not driven by the choice of a specific threshold. Despite this rational, the thresholds may be method and/or population dependent. While these findings demonstrate potential for a microbiome-based diagnostic test, external validation is first needed. Subsequent implementation in clinical laboratories would also require validation with characterized reference samples.

*Enterobacterales* (mainly *Escherichia coli*) remain the major cause of pancreatic infections.^2^^,^^30^ Surprisingly, we did not find an increase in the upper or lower microbiota of patients with infected necrosis, in contrast to other studies that found associations between *Enterobacterales* and complications, such as pancreatic necrosis^11^ and ARDS.^35^ Several factors may explain this. Patients with infected necrosis are generally administered antibiotics directed at *Enterobacterales*, which could suppress their detection in our results. Additionally, *Enterococcus* expansion may competitively suppress *Enterobacterales* in the intestinal microbiota. Furthermore, enteropathogens that invade the bloodstream proliferate at the intestinal mucosa rather than the lumen, meaning that the used luminal sampling method in our study may not fully recapitulate the microbial niche relevant for enteropathogen invasion.

Despite these sampling limitations, decreased gastrointestinal motility and ileus commonly observed in critically ill patients with severe pancreatitis leads to bacterial stasis, overgrowth of colonic-type bacteria in the small intestine and logarithmic increases in pathogen abundance—phenomena particularly evident in ICU patients.^36^^,^^37^ This may partially explain why both saliva and rectal samples captured similar *Enterococcus* expansion patterns, as bacterial redistribution during critical illness potentially reduces the distinction between oral and colonic microbiota. Although endoscopic sampling of the duodenal and small intestinal mucosa would provide more direct insight, such invasive procedures are associated with ethical, logistical and financial hurdles, making them extremely difficult to implement for research or clinical prediction purposes.

Furthermore, our study found that early antibiotic administration was associated with both the risk of infected necrosis and *Enterococcus* colonization. A clear indication for early antibiotic administration was lacking in many cases, consistent with previous findings of our group.^2^ Despite international guidelines recommending against prophylactic antibiotics, early antibiotic use without a documented infection remains prevalent in clinical practice. This likely reflects the difficulty distinguishing systemic inflammation following severe pancreatitis from infection. Several studies showed that prophylactic antibiotics increased the incidence of fungal infections^38-41^; however, this is the first human study that suggests that this practice—besides population-level concerns about antimicrobial resistance—may drive disease progression. As expected, early antibiotic administration was associated with a decrease of alpha diversity and butyrate producing bacteria, indicating a shift towards a disrupted microbiota. This is consistent with a pre-clinical study that showed that prophylactic carbapenem administration to mice undergoing induction of experimental pancreatitis, accelerated mortality due to *Enterococcus gallinarum* bloodstream infection.^42^ Similarly, clindamycin-induced overgrowth and subsequent translocation of enterococci causes extra-intestinal infections in CX3CR1^-/-^ mice.^43^ The mechanistic link between early antibiotic use and infected necrosis follows a plausible pathophysiological sequence: antibiotic administration disrupts the protective commensal microbiome, leading to overgrowth of enterococci that translocate under conditions of inflammation-induced intestinal permeability and seed necrotic pancreatic tissue. While our observational design cannot definitively establish causality, the temporal sequence observed in our cohort, combined with the mentioned preclinical evidence suggests that early antibiotics may directly contribute to, rather than merely associate with, infection risk.

There are a few limitations that need to be taken into consideration when interpreting our results. First, although the initial population is fairly large, infected necrosis is rare and therefore this subgroup is relatively small. Clinically important taxa may therefore not have been detected in this study. Although the direction of effect is clear, the effect estimates should be interpreted with caution as reflected by the wide confidence intervals of the hazard ratios. Similarly, the predominance of cephalosporins among early antibiotic courses precluded meaningful analysis of class-specific effects on microbiota composition, which warrants investigation in larger cohorts. Secondly, 16S rRNA amplicon sequencing was used for profiling of the bacterial microbiota. Other members of the gastrointestinal microbiota (i.e. fungi, bacteriophages and viruses) might play a role in pancreatic infections and remain uninvestigated. Also, using this method, identification of taxa is largely limited to genus-level and does not provide insight on functional aspects, such as metabolism, virulence and antibiotic resistance, which could help determine if, and what, causal role *Enterococcus* plays in progression to infected necrosis. Specifically, we cannot distinguish between *E. faecium* and *E. faecalis*, which differ in pathogenic properties; *E. faecalis* has more virulence factors (cytolysin, aggregation substance, gelatinase), while *E. faecium* harbors more antibiotic resistance, including vancomycin resistance.^44^ This limits both the interpretation of the associations that were identified, and the formulation of species-targeted treatment strategies. For future research, trans-kingdom metagenomics that includes these aspects is preferable. Third, current availability and runtime of metagenomics pipelines are insufficient for implementation in routine clinical practice. Validation of these findings using a method with a short time-to-result for clinical actionability, such as Oxford Nanopore Technologies or the IS-pro method, is warranted.^45^ Fourth, the investigated taxa emerged from hypothesis-driven analyses after untargeted methods yielded no significant results. While this could be considered exploratory, the targeted analysis was based on a priori biological knowledge—enterococci are among the most frequently cultured pathogens from IPN—rather than data-driven selection.

To conclude, gastrointestinal *Enterococcus* expansion, associated with early antibiotic use, preceded infected necrosis by weeks in this prospective multicenter cohort. In clinical practice, monitoring for *Enterococcus* by twice weekly sampling could prompt intensified monitoring, earlier imaging, or (re-)consideration of pre-emptive drainage in patients with established necrotic collections. Furthermore, colonized patients who develop IPN may benefit from empirical antibiotic regimens covering enterococci, which are not covered by the cephalosporins commonly used in this population. Most importantly, our finding that early antibiotic use was associated with increased IPN risk further supports a restrictive antibiotic policy in patients without documented infection. Future trials should evaluate whether targeted interventions in colonized patients—including microbiome-directed therapies and prophylactic strategies that previously failed in unstratified populations—can reduce IPN incidence. External validation is needed to confirm these associations before these findings may be implemented into clinical practice.

## Supplementary Material

POEMA_longitudinal_Supplement_R1_260217_clean.docx POEMA_longitudinal_Supplement_R1_260217_clean.docx

## Data Availability

The 16S rRNA sequencing data have been deposited in the European Nucleotide Archive under accession number PRJEB105316. Clinical data are available from the corresponding author upon reasonable request, subject to ethical approval.

## References

[1] van Dijk SM, Hallensleben NDL, van Santvoort HC, Fockens P, van Goor H, Bruno MJ, Besselink MG. Acute pancreatitis: recent advances through randomised trials. Gut. 2017;66(11):2024–2032. doi: 10.1136/gutjnl-2016-313595. 28838972

[2] Timmerhuis HC, van den Berg FF, Noorda PC, van Dijk SM, van Grinsven J, Sperna Weiland CJ, Umans DS, Mohamed YA, Curvers WL, Bouwense SA, et al. Overuse and misuse of antibiotics and the clinical consequence in necrotizing pancreatitis: an observational multicenter study. Ann Surg. 2023;278(4):e812–e8e9. doi: 10.1097/SLA.0000000000005790. 36728517

[3] Zhou J, Chen W, Liu Y, Qu C, Jiang W, Yin J, Lin J, Mao W, Ye B, Ke L, et al. Trajectories of lymphocyte counts in the early phase of acute pancreatitis are associated with infected pancreatic necrosis. Clin Transl Gastroenterol. 2021;12(9):e00405. doi: 10.14309/ctg.0000000000000405. 34597275 PMC8462575

[4] Ueda T, Takeyama Y, Yasuda T, Shinzeki M, Sawa H, Nakajima T, Ajiki T, Fujino Y, Suzuki Y, Kuroda Y. Immunosuppression in patients with severe acute pancreatitis. J Gastroenterol. 2006;41(8):779–784. doi: 10.1007/s00535-006-1852-8. 16988767

[5] Shen X, Sun J, Ke L, Zou L, Li B, Tong Z. Reduced lymphocyte count as an early marker for predicting infected pancreatic necrosis. BMC Gastroenterol. 2015;15:147. doi: 10.1186/s12876-015-0375-2. 26498708 PMC4620593

[6] Liu S, Szatmary P, Lin JW, Wang Q, Sutton R, Chen L, Huang W, Xia Q. Circulating monocytes in acute pancreatitis. Front Immunol. 2022;13:1062849. doi: 10.3389/fimmu.2022.1062849. 36578487 PMC9791207

[7] Besselink MG, van Santvoort HC, Boermeester MA, Nieuwenhuijs VB, van Goor H, Dejong CHC, Schaapherder AF, Gooszen HG. Timing and impact of infections in acute pancreatitis. Br J Surg. 2009;96(3):267–273. doi: 10.1002/bjs.6447. 19125434

[8] Yu S, Xiong Y, Xu J, Liang X, Fu Y, Liu D, Wu D. Identification of dysfunctional gut microbiota through rectal swab in patients with different severity of acute pancreatitis. Dig Dis Sci. 2020;65(11):3223–3237. doi: 10.1007/s10620-020-06061-4. 32076933

[9] Hu X, Gong L, Zhou R, Han Z, Ji L, Zhang Y, Wu D. Variations in gut microbiome are associated with prognosis of hypertriglyceridemia-associated acute pancreatitis. Biomolecules. 2021;11(5):695. doi: 10.3390/biom11050695. 34066441 PMC8148198

[10] Yu S, Xiong Y, Fu Y, Chen G, Zhu H, Mo X, Wu D, Xu J. Shotgun metagenomics reveals significant gut microbiome features in different grades of acute pancreatitis. Microb Pathog. 2021;154:104849. doi: 10.1016/j.micpath.2021.104849. 33781869

[11] Zou M, Yang Z, Fan Y, Gong L, Han Z, Ji L, Hu X, Wu D. Gut microbiota on admission as predictive biomarker for acute necrotizing pancreatitis. Front Immunol. 2022;13:988326. doi: 10.3389/fimmu.2022.988326. 36105818 PMC9466706

[12] Tan C, Ling Z, Huang Y, Cao Y, Liu Q, Cai T, Yuan H, Li Y, Xu K. Dysbiosis of intestinal microbiota associated with inflammation involved in the progression of acute pancreatitis. Pancreas. 2015;44(6):868–875. doi: 10.1097/MPA.0000000000000355. 25931253

[13] Zhu Y, He C, Li X, Cai Y, Hu J, Liao Y, Zhao J, Xia L, Liu L, Luo C, et al. Gut microbiota dysbiosis worsens the severity of acute pancreatitis in patients and mice. J Gastroenterol. 2019;54(4):347–358. doi: 10.1007/s00535-018-1529-0. 30519748

[14] Ammer-Herrmenau C, Antweiler KL, Asendorf T, Beyer G, Buchholz SM, Cameron S, Capurso G, Damm M, Dang L, Frost F, et al. Gut microbiota predicts severity and reveals novel metabolic signatures in acute pancreatitis. Gut. 2024;73(3):485–495. doi: 10.1136/gutjnl-2023-330987. 38129103 PMC10894816

[15] Pauw HS, Timmerhuis HC, Besselink MG, Issa Y, Bruno M, de Jonge PJF, van Goor H, van Geenen EM, Quispel R, van de Vrie W, et al. Identification of robust associations between admission microbiome profiles and complications of acute pancreatitis. BMJ Open Gastroenterol. 2025;12(1):e001961. doi: 10.1136/bmjgast-2025-001961. PMC12414194 40912695

[16] van den Berg FF, Timmerhuis F, Hugenholtz WJ, Wiersinga JC, Alverdy Y, Issa MG, Besselink H, van Goor MJ, Bruno MA, Boermeester HC, et al. (2021) Prospective, longitudinal study for gut microbiota profiling in patients with acute pancreatitis; the POEMA study protocol: University of Amsterdam.

[17] Elm E, Altman DG, Egger M, Pocock SJ, Gøtzsche PC, Vandenbroucke JP. Strengthening the reporting of observational studies in epidemiology (STROBE) statement: guidelines for reporting observational studies. Brit Med J. 2007;335(7624):806–808. doi: 10.1136/bmj.39335.541782.AD. 17947786 PMC2034723

[18] Banks PA, Bollen TL, Dervenis C, Gooszen HG, Johnson CD, Sarr MG, Tsiotos GG, Vege SS. Classification of acute pancreatitis--2012: revision of the Atlanta classification and definitions by international consensus. Gut. 2013;62(1):102–111. doi: 10.1136/gutjnl-2012-302779. 23100216

[19] Schlebusch S, Graham RMA, Jennison AV, Lassig-Smith MM, Harris PNA, Lipman J, Ó Cuív P, Paterson DL. Standard rectal swabs as a surrogate sample for gut microbiome monitoring in intensive care. BMC Microbiol. 2022;22(1):99. doi: 10.1186/s12866-022-02487-0. 35413802 PMC9004175

[20] Bassis CM, Moore NM, Lolans K, Seekatz AM, Weinstein RA, Young VB, Hayden MK. Comparison of stool versus rectal swab samples and storage conditions on bacterial community profiles. BMC Microbiol. 2017;17(1):78. doi: 10.1186/s12866-017-0983-9. 28359329 PMC5374586

[21] Bansal S, Nguyen JP, Leligdowicz A, Zhang Y, Kain KC, Ricciuto DR, Coburn B. Rectal and naris swabs: practical and informative samples for analyzing the microbiota of critically ill patients. mSphere. 2018;3(3), 10.1128/mSphere.00328-18. PMC6001609 29898981

[22] Edgar RC. Search and clustering orders of magnitude faster than BLAST. Bioinformatics. 2010;26(19):2460–2461. doi: 10.1093/bioinformatics/btq461. 20709691

[23] Wang Q, Garrity GM, Tiedje JM, Cole JR. Naive Bayesian classifier for rapid assignment of rRNA sequences into the new bacterial taxonomy. Appl Environ Microbiol. 2007;73(16):5261–5267. doi: 10.1128/AEM.00062-07. 17586664 PMC1950982

[24] Quast C, Pruesse E, Yilmaz P, Gerken J, Schweer T, Yarza P, Peplies J, Glöckner FO. The SILVA ribosomal RNA gene database project: improved data processing and web-based tools. Nucleic Acids Res. 2013;41(Database issue):D590–D596. doi: 10.1093/nar/gks1219. 23193283 PMC3531112

[25] Lin H, Peddada SD. Analysis of compositions of microbiomes with bias correction. Nat Commun. 2020;11(1):3514. doi: 10.1038/s41467-020-17041-7. 32665548 PMC7360769

[26] Mallick H, Rahnavard A, McIver LJ, Ma S, Zhang Y, Nguyen LH, Tickle TL, Weingart G, Ren B, Schwager EH, et al. Multivariable association discovery in population-scale meta-omics studies. PLoS Comput Biol. 2021;17(11):e1009442. doi: 10.1371/journal.pcbi.1009442. 34784344 PMC8714082

[27] Kullberg RFJ, Wikki I, Haak BW, Kauko A, Galenkamp H, Peters-Sengers H, Butler JM, Havulinna AS, Palmu J, McDonald D, et al. Association between butyrate-producing gut bacteria and the risk of infectious disease hospitalisation: results from two observational, population-based microbiome studies. Lancet Microbe. 2024;5(9):100864. doi: 10.1016/S2666-5247(24)00079-X. 38909617

[28] Talukdar R, Nechutova H, Clemens M, Vege SS. Could rising BUN predict the future development of infected pancreatic necrosis? Pancreatology. 2013;13(4):355–359. doi: 10.1016/j.pan.2013.05.003. 23890133

[29] Fan N, Hu Y, Shen H, Liu S, Zhao G, Sun L, Li C, Wang J, Cui Y. Compositional and drug-resistance profiling of pathogens in patients with severe acute pancreatitis: a retrospective study. BMC Gastroenterol. 2020;20(1):405. doi: 10.1186/s12876-020-01563-x. 33261570 PMC7709241

[30] Guo Q, Li A, Xia Q, Liu X, Tian B, Mai G, Huang Z, Chen G, Tang W, Jin X, et al. The role of organ failure and infection in necrotizing pancreatitis: a prospective study. Ann Surg. 2014;259(6):1201–1207. doi: 10.1097/SLA.0000000000000264. 24169172

[31] Shepard BD, Gilmore MS. Differential expression of virulence-related genes in *Enterococcus faecalis* in response to biological cues in serum and urine. Infect Immun. 2002;70(8):4344–4352. doi: 10.1128/IAI.70.8.4344-4352.2002. 12117944 PMC128128

[32] Archambaud C, Derre-Bobillot A, Lapaque N, Rigottier-Gois L, Serror P. Intestinal translocation of enterococci requires a threshold level of enterococcal overgrowth in the lumen. Sci Rep. 2019;9(1):8926. doi: 10.1038/s41598-019-45441-3. 31222056 PMC6586816

[33] van den Berg FF, van Dalen D, Hyoju SK, van Santvoort HC, Besselink MG, Wiersinga WJ, Zaborina O, Boermeester MA, Alverdy J. Western-type diet influences mortality from necrotising pancreatitis and demonstrates a central role for butyrate. Gut. 2021;70(5):915–927. doi: 10.1136/gutjnl-2019-320430. 32873697 PMC7917160

[34] Li Q, Wang C, Tang C, Zhao X, He Q, Li J. Identification and characterization of blood and neutrophil-associated microbiomes in patients with severe acute pancreatitis using next-generation sequencing. Front Cell Infect Microbiol. 2018;8:5. doi: 10.3389/fcimb.2018.00005. 29423379 PMC5790034

[35] Hu X, Han Z, Zhou R, Su W, Gong L, Yang Z, Song X, Zhang S, Shu H, Wu D. Altered gut microbiota in the early stage of acute pancreatitis were related to the occurrence of acute respiratory distress syndrome. Front Cell Infect Microbiol. 2023;13:1127369. doi: 10.3389/fcimb.2023.1127369. 36949815 PMC10025409

[36] Cho NA, Strayer K, Dobson B, McDonald B. Pathogenesis and therapeutic opportunities of gut microbiome dysbiosis in critical illness. Gut Microbes. 2024;16(1):2351478. doi: 10.1080/19490976.2024.2351478. 38780485 PMC11123462

[37] McDonald D, Ackermann G, Khailova L, Baird C, Heyland D, Kozar R, Lemieux M, Derenski K, King J, Vis-Kampen C, et al. Extreme dysbiosis of the microbiome in critical illness. mSphere. 2016;1(4). doi: 10.1128/mSphere.00199-16. PMC5007431 27602409

[38] Horibe M, Sanui M, Sasaki M, Honda H, Ogura Y, Namiki S, Sawano H, Goto T, Ikeura T, Takeda T, et al. Impact of antimicrobial prophylaxis for severe acute pancreatitis on the development of invasive candidiasis: a large retrospective multicenter cohort study. Pancreas. 2019;48(4):537–543. doi: 10.1097/MPA.0000000000001269. 30946245

[39] Maravi-Poma E, Gener J, Alvarez-Lerma F, Maraví-Poma E, Olaechea P, Blanco A, Domínguez-Muñoz JE. Early antibiotic treatment (prophylaxis) of septic complications in severe acute necrotizing pancreatitis: a prospective, randomized, multicenter study comparing two regimens with imipenem-cilastatin. Intensive Care Med. 2003;29(11):1974–1980. doi: 10.1007/s00134-003-1956-z. 14551680

[40] Schwender BJ, Gordon SR, Gardner TB. Risk factors for the development of intra-abdominal fungal infections in acute pancreatitis. Pancreas. 2015;44(5):805–807. doi: 10.1097/MPA.0000000000000334. 25872170 PMC4464973

[41] Xue P, Deng LH, Zhang ZD, Yang X, Wan M, Song B, Xia Q. Effect of antibiotic prophylaxis on acute necrotizing pancreatitis: results of a randomized controlled trial. J Gastroenterol Hepatol. 2009;24(5):736–742. doi: 10.1111/j.1440-1746.2008.05758.x. 19220676

[42] Soares FS, Amaral FC, Silva NLC, Valente MR, Santos LKR, Yamashiro LH, Scheffer MC, Castanheira FVES, Ferreira RG, Gehrke L, et al. Antibiotic-induced pathobiont dissemination accelerates mortality in severe experimental pancreatitis. Front Immunol. 2017;8:1890–1890. doi: 10.3389/fimmu.2017.01890. 29375557 PMC5770733

[43] Archambaud C, Derré-Bobillot A, Lapaque N, Rigottier-Gois L, Serror P. Intestinal translocation of enterococci requires a threshold level of enterococcal overgrowth in the lumen. NatSR. 2019;9(1):8926. doi: 10.1038/s41598-019-45441-3. PMC6586816 31222056

[44] Arias CA, Murray BE. The rise of the *Enterococcus*: beyond vancomycin resistance. Nat Rev Microbiol. 2012;10(4):266–278. doi: 10.1038/nrmicro2761. 22421879 PMC3621121

[45] Budding AE, Grasman ME, Lin F, Bogaards JA, Soeltan‐Kaersenhout DJ, Vandenbroucke‐Grauls CMJE, Van Bodegraven AA, Savelkoul PHM. IS-pro: high-throughput molecular fingerprinting of the intestinal microbiota. FASEB J. 2010;24(11):4556–4564. doi: 10.1096/fj.10-156190. 20643909

