# Supplementary material for: Antibiotic-associated Enterococcus expansion in the gastrointestinal tract precedes infected necrosis in acute necrotizing pancreatitis
Source: Gut Microbes. 2026 May 15;18(1):2670039. doi: 10.1080/19490976.2026.2670039 (PMC13182963; doi:10.1080/19490976.2026.2670039)
Supplement: POEMA_longitudinal_Supplement_R1_260217_clean.docx [file KGMI_A_2670039_SM4251.docx]

**Table of Contents**

| **Item** | **Page** |
| --- | --- |
| **Supplementary Methods** | 2 |
| DNA extraction and 16S rRNA gene sequencing | 2 |
| Bioinformatic processing and taxonomic assignment | 2 |
| Detailed statistical methods and model specifications | 2 |
| **Supplementary Figures** | 4 |
| Supplementary Figure 1. Flowchart of study | 4 |
| Supplementary Figure 2. Histogram of total acquired reads per sample type | 5 |
| Supplementary Figure 3. Relative abundance trajectories | 6 |
| Supplementary Figure 4. Average microbiota composition at Family-level | 8 |
| Supplementary Figure 5. Correlation between Inverse Simpson score and Enterococcus | 9 |
| Supplementary Figure 6. Pancreatic isolates and relative abundance | 10 |
| Supplementary Figure 7. Landmark receiver operating characteristic curves comparing rectal Enterococcus abundance and CRP for discrimination of infected pancreatic necrosis. | 11 |
|  |  |
| **Supplementary Tables** | 12 |
| Supplementary Table 1. LME model ANOVA for alpha diversity: infection status | 12 |
| Supplementary Table 2. LME model coefficients for alpha diversity: infection status | 12 |
| Supplementary Table 3. LME model results: infection status on PCoA trajectories | 12 |
| Supplementary Table 4. Pancreatic cultures from drainage/necrosectomy | 13 |
| Supplementary Table 5. Zero-inflated mixed model: count analysis | 13 |
| Supplementary Table 6. Zero-inflated mixed model: excess zero analysis | 14 |
| Supplementary Table 7. Time-dependent Cox regression by sample type and covariate adjustment. | 14 |
| Supplementary Table 8. Individual patient characteristics | 15 |
| Supplementary Table 9. Antibiotic administration | 16 |
| Supplementary Table 10. LME model ANOVA for alpha diversity: early antibiotics | 16 |
| Supplementary Table 11. LME model coefficients for alpha diversity: early antibiotics | 16 |
| Supplementary Table 12. LME model results: early antibiotics on PCoA trajectories | 16 |
| Supplementary Table 13. LME model ANOVA: early antibiotics on butyrate-producers | 17 |
| Supplementary Table 14. LME model coefficients: early antibiotics on butyrate-producers | 17 |
| **References** | 17 |

**Extended Methods**

**DNA extraction and 16S rRNA gene sequencing**

DNA was extracted from rectal swabs and saliva using a modification of a previously described protocol (method 5). ^1,2^ The storage medium was centrifuged and the pellet resuspended in 300 µL STAR Buffer and 30 µL proteinase K. The suspension was added to a beat beating tube together with the cotton tip in case of a rectal swab, followed by the beat beating protocol. DNA was purified using the Maxwell RSC Whole Blood DNA kit with the Maxwell RSC Instrument. Negative controls, including water controls processed through the entire workflow, were included to assess potential contamination. 16S rRNA gene amplicons were generated using a single step PCR protocol targeting the V3-V4 region. ^3^ PCR products were purified using Ampure XP beads and purified products were equimolar pooled. The libraries were sequenced using an Illumina MiSeq platform using V3 chemistry with 2x251 cycles. Samples were randomly distributed across two sequencing batches to control for potential batch effects.

**Bioinformatic processing and taxonomic assignment**

Forward and reverse reads were truncated to 240 and 210 bases respectively and merged using USEARCH. ^4^ Merged reads that did not pass the Illumina chastity filter, had an expected error rate higher than 2, or were shorter than 380 bases were filtered. Amplified Sequence Variants (ASVs) were inferred for each sample individually with a minimum abundance of 4 reads. ^5^ Unfiltered reads were than mapped against the collective ASV set to determine the abundances. Taxonomy was assigned using the RDP classifier^6^ and SILVA 16S ribosomal database V132^7^. Samples with less than 10,000 sequences were removed to filter out low quality samples. ASVs with a relative abundance <0.1% per sample were removed to filter out spurious taxa. ^8^

**Detailed statistical methods and model specifications**

**Diversity analyses**: Rarefaction was applied for alpha- and beta diversity analysis to balance the differences in reads, and rarefaction depth was based on visual inspection of the rarefaction curve. Alpha diversity, the within-sample diversity, was quantified using the inverse Simpson diversity index and analysed separately for rectal swab and saliva samples. Longitudinal changes were modelled using linear mixed-effects models with the lmer() function from the lme4 package in R, specifying infection status, time (day), and their interaction as fixed effects, with the subject as a random intercept to account for repeated measures. Statistical significance of fixed effects was assessed using Type II Wald chi-square tests via the Anova() function (car package), and post-hoc comparisons at specific timepoints were conducted using estimated marginal means (emmeans package). Model assumptions were verified through residual plot inspection.

For beta-diversity (between-sample diversity), Bray-Curtis distances were calculated from relative abundances and subjected to principal coordinates analysis (PCoA) using pcoa() (ape v5.5). PCoA axes were retained based on eigenvalue magnitude and cumulative variance explained. Linear mixed-effects models were fitted to PCoA coordinate values using lmer() (lme4 v1.1-27.1). Models included fixed effects for infection status, time (days from admission), and their interaction, with participant-specific random intercepts and slopes. Statistical significance was assessed using lmerTest (v3.1-3) with α=0.05.

**Differential abundance testing:** Ancom-BC2^9^ and MaAsLin2 ^10^ with recent antibiotic treatment and infection status as fixed effects, and standard settings were used for differential abundance testing per timepoint. For longitudinal differential abundance, the study subject was used as random effects. FDR-adjusted p-values (q values) less than 0.05 were considered statistically significant. Current antibiotic treatment was (arbitrarily) pre-defined as antibiotic administration for at least 2 consecutive days in the week before sample collection. For targeted longitudinal differential abundance analysis of taxa, zero-inflated negative binomial mixed-effects models were fitted using the glmmTMB() function from the glmmTMB R package to handle excess zeros and account for repeated measurements within participants. Enterobacterales in saliva samples exhibited excessive sparsity that precluded model convergence and was therefore excluded from the analysis.

**Butyrate-producer analysis:** Linear mixed-effects models (lmer function, lme4 package) were used for longitudinal butyrate-producer relative abundance, with early antibiotic exposure group, time, and their interaction as fixed effects, and subject as a random effect. Post-hoc timepoint comparisons were performed using estimated marginal means (emmeans package).

**Cox regression specifications:** Right-censoring was applied at the minimum follow-up time (90 days), or death. Optimal cutoffs for colonization were determined based on visual inspection of the relative abundance data. BMI, stool consistency and alcohol use were dichotomized as previously done. ^11^ Missing values in the questionnaire were imputed using multiple imputation by chained equations (R package 'mice' v3.16.0), generating 5 imputed datasets. The imputation model included 17 baseline auxiliary variables: demographics (age, gender, height, weight), medical history (cardiovascular, pulmonary, renal, diabetes, smoking, alcohol use), admission severity scores (APACHE-II, Imrie, CRP), and disease aetiology. Missing data patterns were assessed using R packages 'VIM' and 'naniar'. Pearson correlation was used for correlation testing.

**Landmark analyses:** Performed at days 3, 7, 10, 14, 17, and 21 after admission. At each landmark, patients still at risk were included with marker values carried forward from the most recent assessment (last observation carried forward). Time-dependent AUC was estimated at a 4-week prediction horizon using inverse probability of censoring weighting, implemented in the timeROC R package. ^12^ CRP (mg/L) was evaluated as clinical comparator using the most recent measurement before each landmark. Both markers were analysed as continuous variables without covariate adjustment, each on their maximum available sample.

**Results**

**Figure S1**Flowchart of study


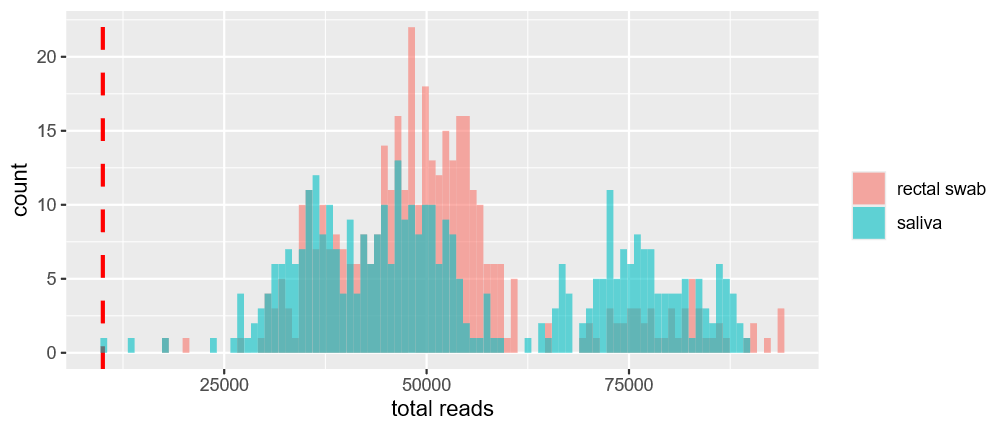


**Figure S2**Histogram of total acquired reads per sample type


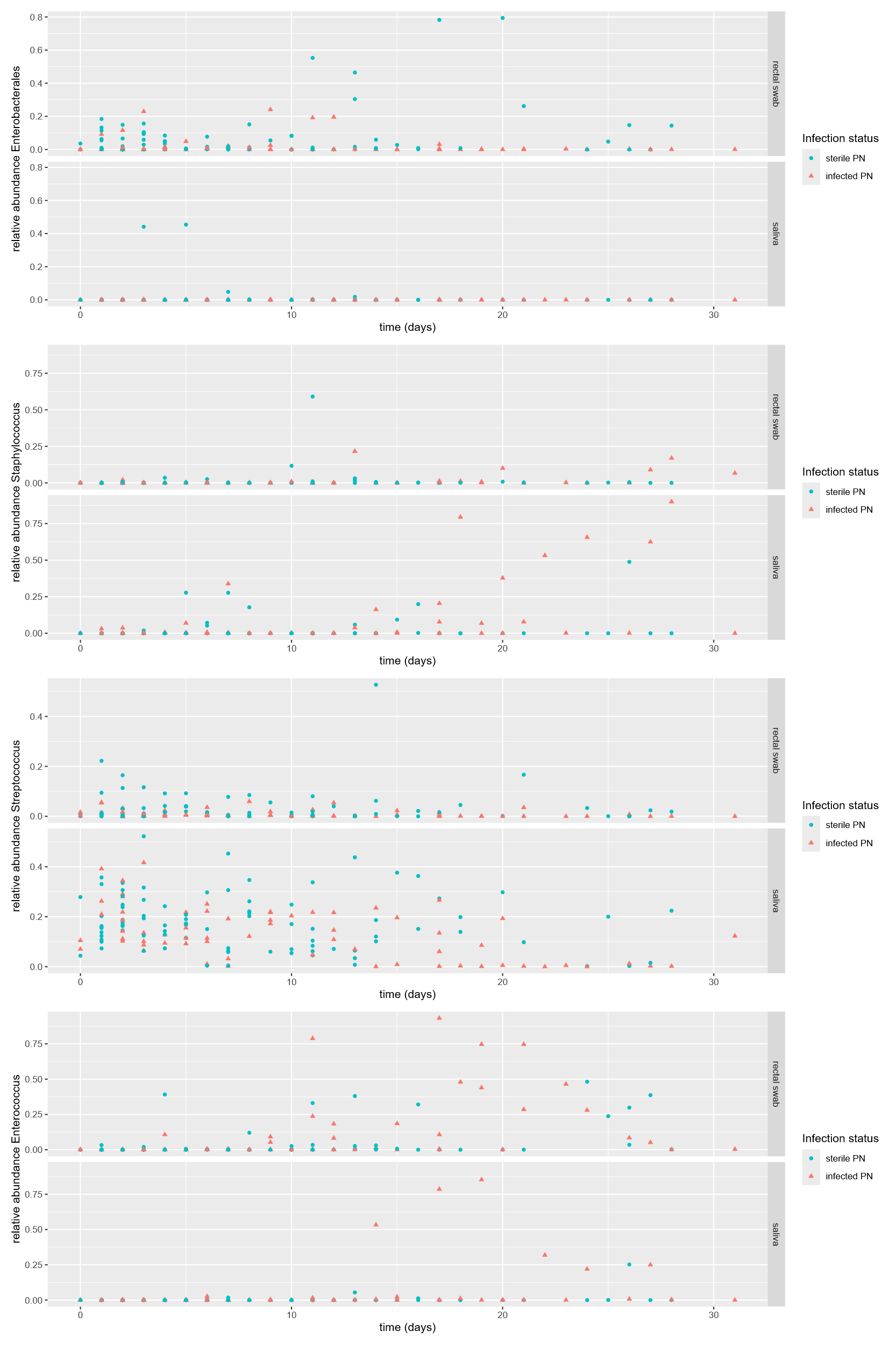


**Figure S3**

Relative abundance trajectories of Enterobacterales (*Citrobacter*, *Escherichia/Shigella*, *Enterobacter*, *Klebsiella*, *Raoultella*, *Morganella*, *Proteus*), *Staphylococcus*, *Streptococcus* and *Enterococcus* in the sterile and infected (peri-)pancreatic necrosis groups.


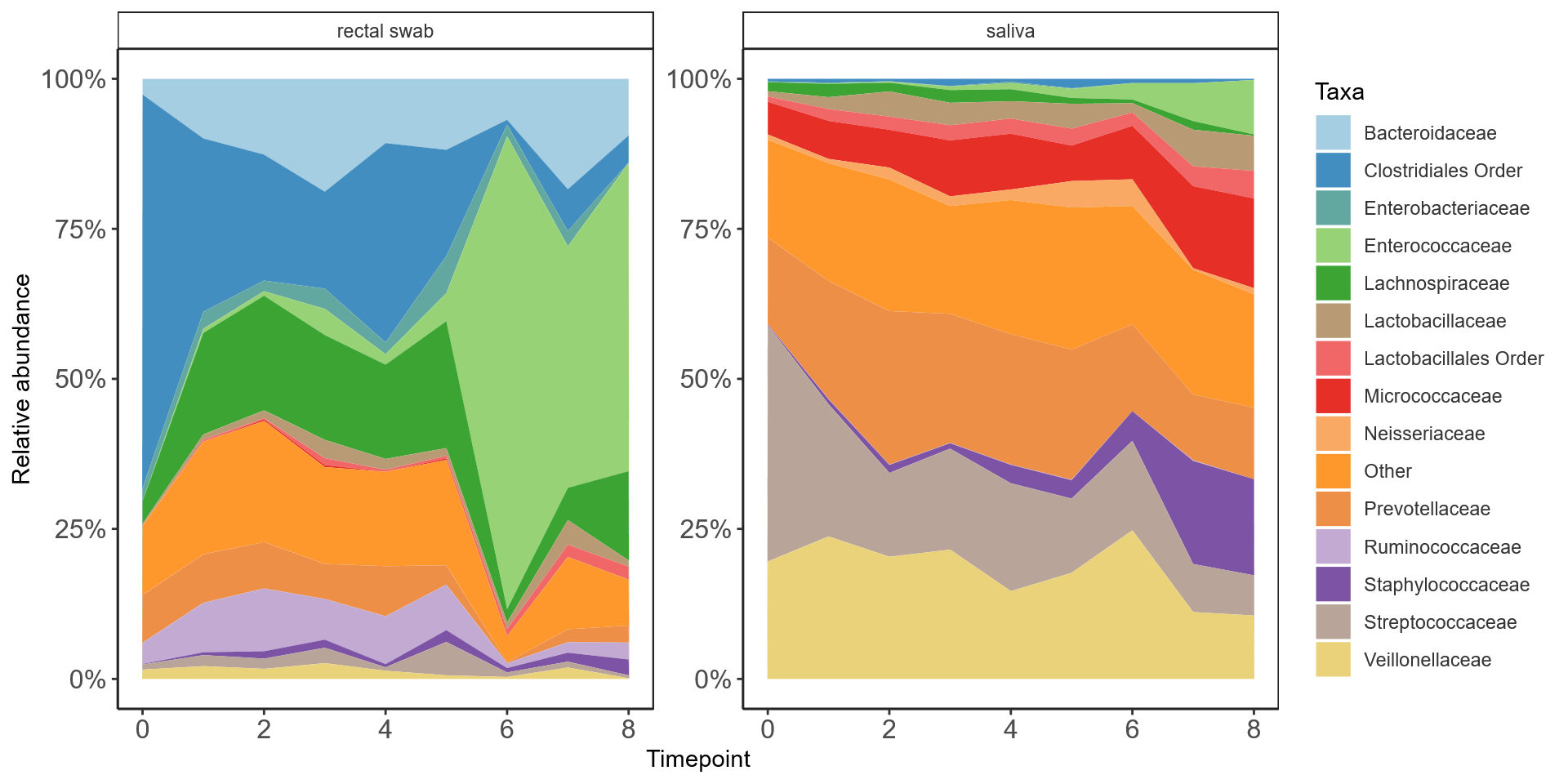


**Figure S4**Average microbiota composition at Family-level across timepoints.


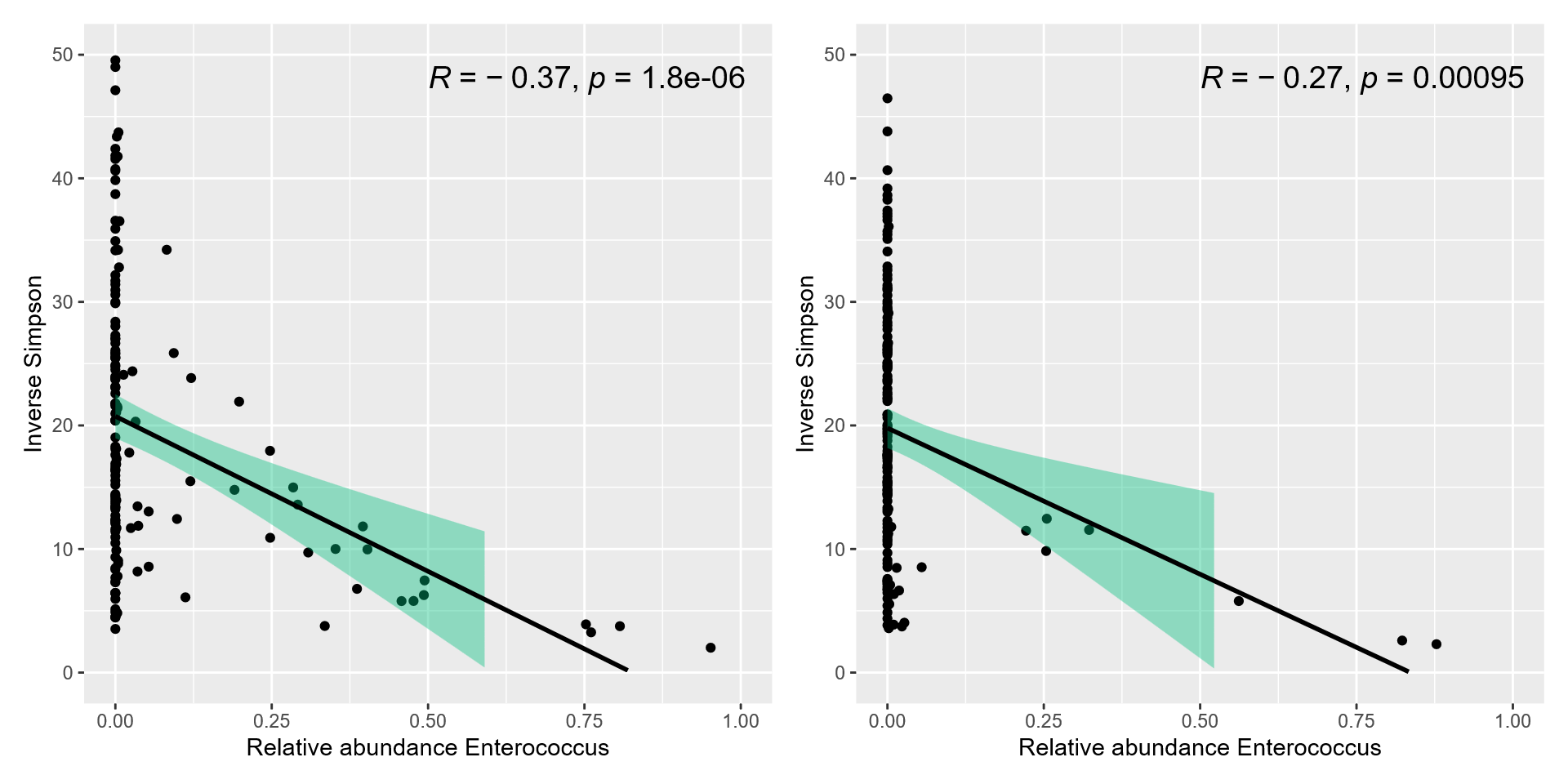


**Figure S5**Correlation between Inverse Simpson score and relative abundance of rectal (left) and salivary (right) *Enterococcus* (left). Pearson correlation was used for correlation testing.


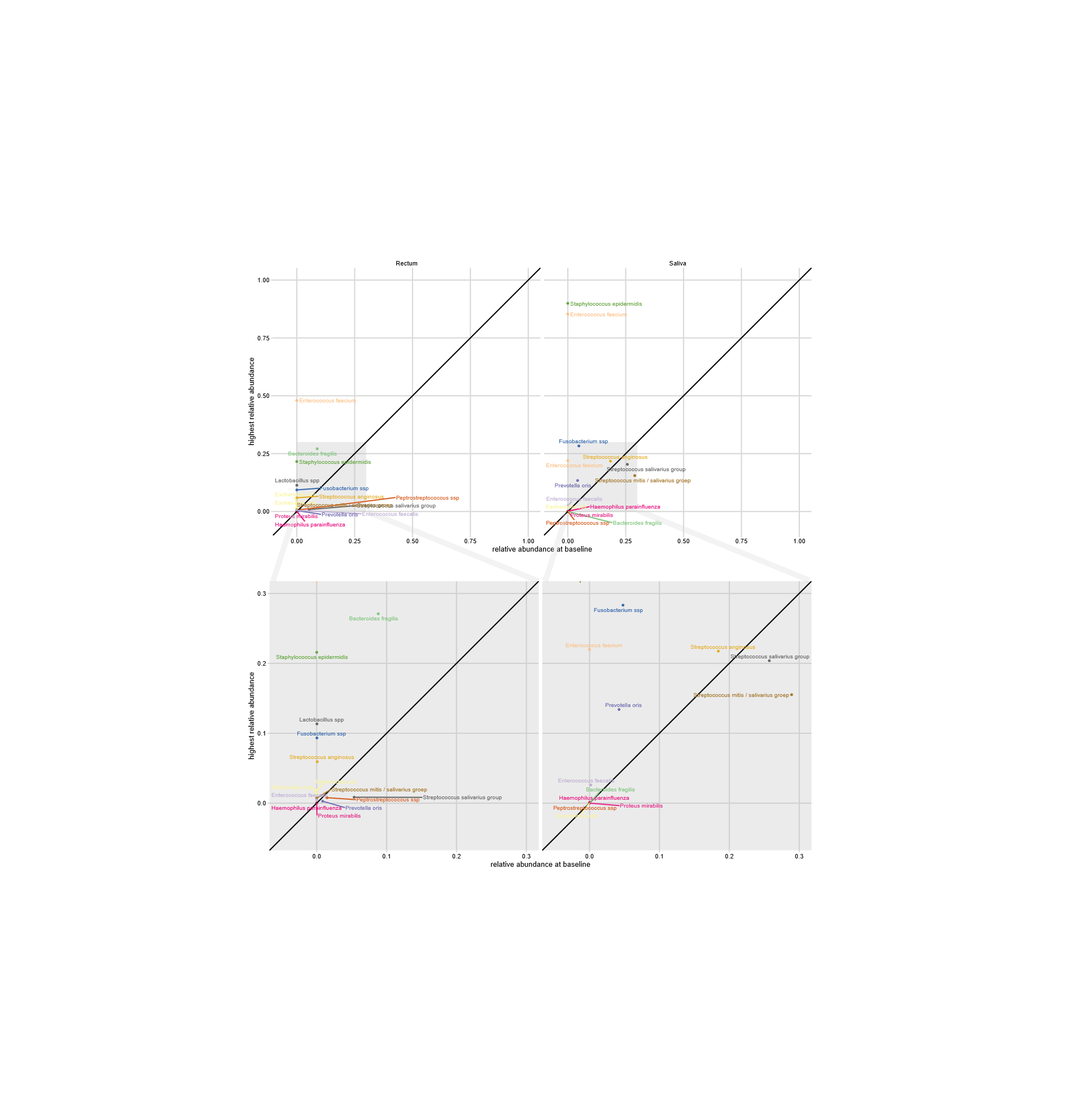


**Figure S6**Pancreatic isolates and relative abundance of the corresponding taxa (Genus-level) in saliva (right) and rectum samples (left). Relative abundance at baseline (x-axis) and the highest during admission are displayed.


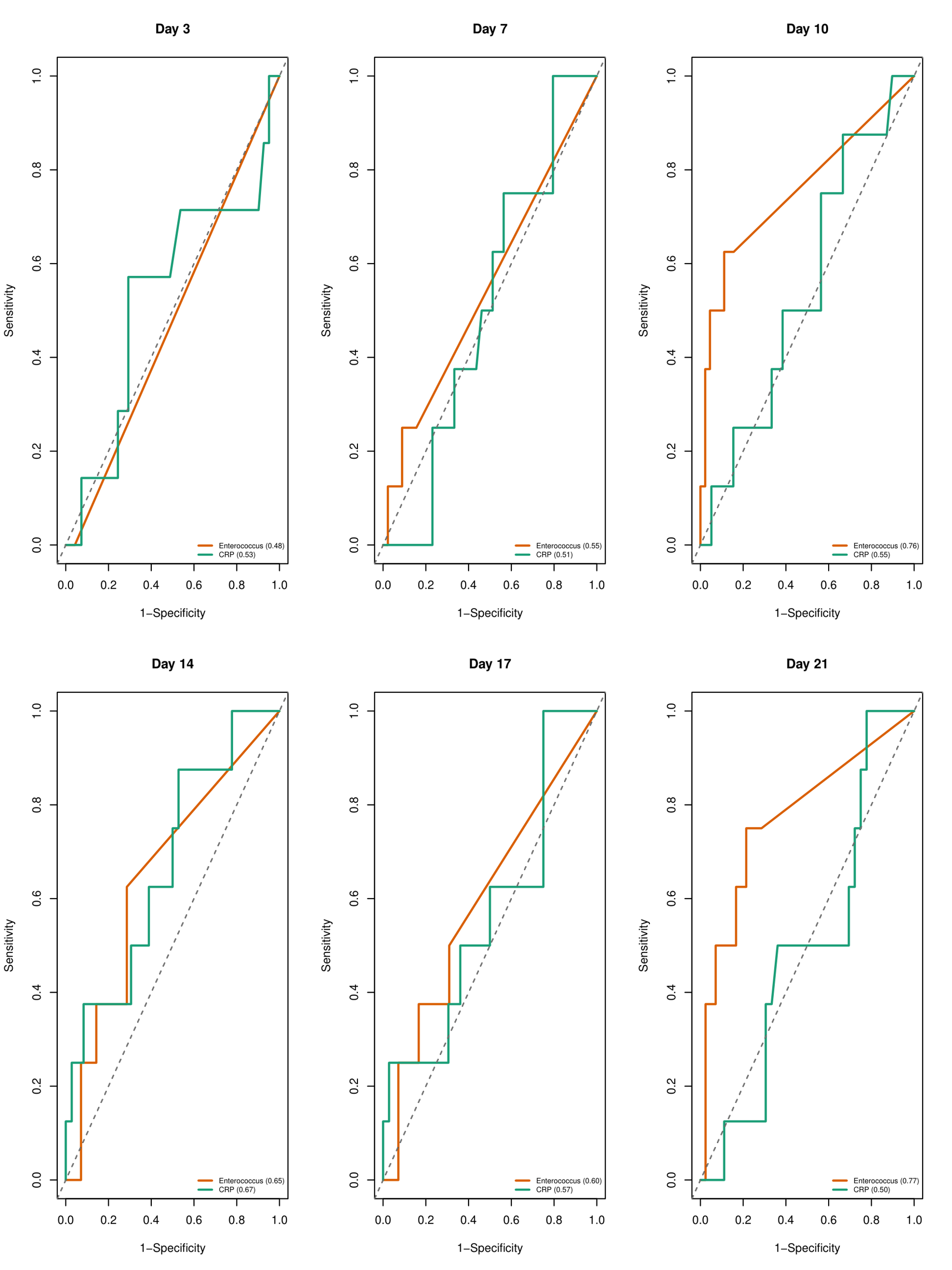


**Figure S7 Landmark receiver operating characteristic curves comparing rectal Enterococcus abundance and CRP for discrimination of infected pancreatic necrosis.**

Each panel shows the ROC curve at a successive landmark day for prediction of infected necrosis within 28 days. AUC values are shown in parentheses. Marker values reflect last observation carried forward.

| **Table S1 Linear mixed-effects model ANOVA for microbiome alpha diversity: infection status** | | | | |
| --- | --- | --- | --- | --- |
| **material** | **effect** | **chisq** | **df** | **p_value** |
| rectal_swab | group | 0.271 | 1 | 0.603 |
| rectal_swab | day | 9.837 | 1 | 0.002 |
| rectal_swab | group:day | 0.465 | 1 | 0.496 |
| saliva | group | 0.286 | 1 | 0.593 |
| saliva | day | 8.647 | 1 | 0.003 |
| saliva | group:day | 0.125 | 1 | 0.723 |

| **Table S2 Linear mixed-effects model model coefficients for microbiome alpha diversity: infection status** | | | | | | | |
| --- | --- | --- | --- | --- | --- | --- | --- |
| **material** | **effect** | **term** | **estimate** | **std.error** | **statistic** | **df** | **p.value** |
| rectal_swab | fixed | (Intercept) | 31.38967 | 2.813342 | 11.15743 | 127.2709 | <0.001 |
| rectal_swab | fixed | groupInfected | 0.492015 | 4.465343 | 0.110185 | 121.8902 | 0.912 |
| rectal_swab | fixed | day | -0.46247 | 0.264525 | -1.74832 | 140.0112 | 0.083 |
| rectal_swab | fixed | groupInfected:day | -0.25617 | 0.37584 | -0.68158 | 147.9492 | 0.497 |
| saliva | fixed | (Intercept) | 37.72261 | 3.646621 | 10.34454 | 128.4148 | <0.001 |
| saliva | fixed | groupInfected | -0.97933 | 5.776134 | -0.16955 | 123.8471 | 0.866 |
| saliva | fixed | day | -0.6144 | 0.343855 | -1.7868 | 147.485 | 0.076 |
| saliva | fixed | groupInfected:day | -0.16918 | 0.47779 | -0.35409 | 147.9995 | 0.724 |

| **Table S3 Linear mixed-effects model results: infection status on microbiome PCoA trajectories** | | | | | | | |
| --- | --- | --- | --- | --- | --- | --- | --- |
| **Material** | **PCoA_Axis** | **Variance_Explained** | **Term** | **Estimate** | **Std_Error** | **t_value** | **P_value** |
| rectal swab | PCo1 | 25.442 | (Intercept) | -0.168 | 0.053 | -3.182 | 0.002 |
| rectal swab | PCo1 | 25.442 | Infection status | 0.015 | 0.068 | 0.228 | 0.820 |
| rectal swab | PCo1 | 25.442 | Day | 0.017 | 0.004 | 4.141 | <0.001 |
| rectal swab | PCo1 | 25.442 | Infection status:Day | 0.001 | 0.006 | 0.215 | 0.830 |
| rectal swab | PCo2 | 11.982 | (Intercept) | 0.080 | 0.037 | 2.165 | 0.032 |
| rectal swab | PCo2 | 11.982 | Infection status | 0.018 | 0.048 | 0.375 | 0.708 |
| rectal swab | PCo2 | 11.982 | Day | -0.012 | 0.003 | -4.136 | <0.001 |
| rectal swab | PCo2 | 11.982 | Infection status:Day | 0.003 | 0.004 | 0.776 | 0.439 |
| rectal swab | PCo3 | 7.176 | (Intercept) | -0.039 | 0.033 | -1.201 | 0.233 |
| rectal swab | PCo3 | 7.176 | Infection status | 0.001 | 0.042 | 0.032 | 0.974 |
| rectal swab | PCo3 | 7.176 | Day | 0.006 | 0.002 | 2.579 | 0.011 |
| rectal swab | PCo3 | 7.176 | Infection status:Day | -0.004 | 0.003 | -1.185 | 0.238 |
| rectal swab | PCo4 | 6.320 | (Intercept) | -0.023 | 0.028 | -0.821 | 0.413 |
| rectal swab | PCo4 | 6.320 | Infection status | -0.002 | 0.037 | -0.064 | 0.949 |
| rectal swab | PCo4 | 6.320 | Day | 0.000 | 0.002 | 0.170 | 0.865 |
| rectal swab | PCo4 | 6.320 | Infection status:Day | 0.005 | 0.003 | 1.583 | 0.115 |
| saliva | PCo1 | 24.124 | (Intercept) | -0.135 | 0.043 | -3.117 | 0.002 |
| saliva | PCo1 | 24.124 | Infection status | 0.018 | 0.056 | 0.324 | 0.747 |
| saliva | PCo1 | 24.124 | Day | 0.018 | 0.003 | 5.448 | <0.001 |
| saliva | PCo1 | 24.124 | Infection status:Day | -0.007 | 0.005 | -1.582 | 0.116 |
| saliva | PCo2 | 17.159 | (Intercept) | -0.066 | 0.039 | -1.686 | 0.094 |
| saliva | PCo2 | 17.159 | Infection status | 0.025 | 0.050 | 0.507 | 0.613 |
| saliva | PCo2 | 17.159 | Day | 0.009 | 0.003 | 3.197 | 0.002 |
| saliva | PCo2 | 17.159 | Infection status:Day | -0.009 | 0.004 | -2.160 | 0.032 |
| saliva | PCo3 | 9.952 | (Intercept) | -0.010 | 0.031 | -0.316 | 0.753 |
| saliva | PCo3 | 9.952 | Infection status | -0.061 | 0.039 | -1.560 | 0.122 |
| saliva | PCo3 | 9.952 | Day | 0.001 | 0.002 | 0.297 | 0.767 |
| saliva | PCo3 | 9.952 | Infection status:Day | 0.008 | 0.003 | 2.570 | 0.011 |
| saliva | PCo4 | 8.264 | (Intercept) | -0.022 | 0.029 | -0.768 | 0.444 |
| saliva | PCo4 | 8.264 | Infection status | 0.026 | 0.037 | 0.697 | 0.487 |
| saliva | PCo4 | 8.264 | Day | 0.002 | 0.002 | 0.985 | 0.326 |
| saliva | PCo4 | 8.264 | Infection status:Day | -0.002 | 0.003 | -0.489 | 0.626 |

| **Table S4 Pancreatic cultures from 147 drainage or necrosectomy procedures. Adapted from Timmerhuis et al.** | | | |
| --- | --- | --- | --- |
| **Enterococcaceae** | | **69** | **46.9%** |
|  | E. faecium | 47 |  |
|  | E. faecalis | 17 |  |
|  | E. avium | 2 |  |
|  | E. hirae | 1 |  |
|  | Enterococcus species (undefined) | 2 |  |
| **Staphylococcaceae** | | **32** | **21.8%** |
|  | S. aureus | 12 |  |
|  | Coagulase-negative staphylococci | 19 |  |
|  | Staphylococcus species (undefined) | 1 |  |
| **Streptococcaceae** | | **25** | **17.0%** |
|  | S. mitis | 1 |  |
|  | S. milleri | 5 |  |
|  | S. oralis | 2 |  |
|  | S. anginosus | 5 |  |
|  | S. parasanguinis | 2 |  |
|  | S. salivarius | 3 |  |
|  | S. gordonii | 1 |  |
|  | S. dysgalactiae | 2 |  |
|  | Streptococcus species (undefined) | 4 |  |
| **Enterobacteriaceae** | | **77** | **52.4%** |
|  | C. freundii | 4 |  |
|  | E. coli | 48 |  |
|  | E. cloacae complex | 8 |  |
|  | E. aerogenes | 2 |  |
|  | K. pneumoniae | 5 |  |
|  | K. oxytoca | 7 |  |
|  | R. ornithinolytica | 2 |  |
|  | Citrobacter species | 1 |  |
| **Morganellaceae** | | **10** | **6.8%** |
|  | M. morganii | 3 |  |
|  | P. mirabilis | 6 |  |
|  | P. vulgaris | 1 |  |

| **Table S5 Zero-inflated mixed model: count analysis** | | | | | | |
| --- | --- | --- | --- | --- | --- | --- |
| **Taxa** | **Material** | **Parameter** | **Estimate** | **Std_Error** | **Z_value** | **P_value** |
| Enterococcus | Saliva | Intercept | 4.678 | 2.673 | 1.750 | 0.080 |
|  | Saliva | Infected_necrosis_YNInfection | -2.410 | 2.681 | -0.899 | 0.369 |
|  | Saliva | time_centered | 0.443 | 0.373 | 1.188 | 0.235 |
|  | Rectal | Intercept | 5.636 | 0.508 | 11.085 | <0.001 |
|  | Rectal | Infected_necrosis_YNInfection | 0.876 | 0.758 | 1.156 | 0.248 |
|  | Rectal | time_centered | 0.099 | 0.041 | 2.424 | 0.015 |
| Staphylococcus | Saliva | Intercept | 7.223 | 0.356 | 20.317 | <0.001 |
|  | Saliva | Infected_necrosis_YNInfection | -0.397 | 0.469 | -0.847 | 0.397 |
|  | Saliva | time_centered | 0.103 | 0.028 | 3.718 | 0.000 |
|  | Rectal | Intercept | 4.511 | 1.024 | 4.405 | 0.000 |
|  | Rectal | Infected_necrosis_YNInfection | -2.109 | 0.984 | -2.143 | 0.032 |
|  | Rectal | time_centered | 0.242 | 0.087 | 2.771 | 0.006 |
| Streptococcus | Saliva | Intercept | 7.468 | 0.094 | 79.430 | <0.001 |
|  | Saliva | Infected_necrosis_YNInfection | -0.215 | 0.149 | -1.450 | 0.148 |
|  | Saliva | time_centered | -0.029 | 0.011 | -2.690 | 0.007 |
|  | Rectal | Intercept | 5.567 | 0.259 | 21.457 | <0.001 |
|  | Rectal | Infected_necrosis_YNInfection | -0.795 | 0.377 | -2.111 | 0.035 |
|  | Rectal | time_centered | -0.022 | 0.022 | -0.992 | 0.321 |
| Enterobacterales | Rectal | Intercept | 6.214 | 0.285 | 21.819 | <0.001 |
|  | Rectal | Infected_necrosis_YNInfection | -0.479 | 0.462 | -1.037 | 0.300 |
|  | Rectal | time_centered | 0.004 | 0.029 | 0.129 | 0.897 |

| **Table S6 Zero-inflation mixed model: Excess zero analysis** | | | | | | |
| --- | --- | --- | --- | --- | --- | --- |
| **Taxa** | **Material** | **Parameter** | **Estimate** | **Std_Error** | **Z_value** | **P_value** |
| Enterococcus | Saliva | Intercept | 4.721 | 1.373 | 3.439 | 0.001 |
|  | Saliva | Infected_necrosis_YNInfection | -7.471 | 1.262 | -5.919 | <0.001 |
|  | Rectal | Intercept | 0.786 | 0.285 | 2.761 | 0.006 |
|  | Rectal | Infected_necrosis_YN | -0.272 | 0.396 | -0.686 | 0.493 |
| Staphylococcus | Saliva | Intercept | 3.843 | 1.333 | 2.883 | 0.004 |
|  | Saliva | Infected_necrosis_YN | -1.276 | 1.041 | -1.225 | 0.221 |
|  | Rectal | Intercept | -1.757 | 0.752 | -2.336 | 0.020 |
|  | Rectal | Infected_necrosis_YNInfection | -1.370 | 0.770 | -1.780 | 0.075 |
| Streptococcus | Saliva | Intercept | -9.265 | 3.172 | -2.921 | 0.003 |
|  | Saliva | Infected_necrosis_YN | 1.446 | 2.913 | 0.496 | 0.620 |
|  | Rectal | Intercept | -1.026 | 0.301 | -3.412 | 0.001 |
|  | Rectal | Infected_necrosis_YN | 0.866 | 0.435 | 1.992 | 0.046 |

| **Table S7 Time-dependent Cox regression for the association between Enterococcus colonization and infected pancreatic necrosis by sample type and covariate adjustment.** | | | | | | |
| --- | --- | --- | --- | --- | --- | --- |
| **Covariate set** | **Sample type** | **Exposure definition** | **Exposed (n)** | **Events** | **HR (95% CI)** | **P-value** |
| **Biliary etiology, prior antibiotics, APACHE-II** | **Salivary** | Detected (>0%) | 9 | 6 | 5.37 (1.72–16.79) | 0.004 |
|  |  | ≥1% | 5 | 3 | 2.72 (0.58–12.91) | 0.207 |
|  |  | ≥2.5% | 4 | 3 | 3.20 (0.66–15.42) | 0.147 |
|  |  | ≥5% | 3 | 2 | 3.51 (0.71–17.43) | 0.125 |
|  |  | ≥7.5% | 3 | 2 | 3.56 (0.72–17.70) | 0.121 |
|  |  | ≥10% | 3 | 2 | 3.56 (0.72–17.70) | 0.121 |
|  |  | Continuous (log₁₀)* |  |  | 1.80 (1.04–3.10) | 0.035 |
|  |  | Inverse Simpson† |  |  | 0.99 (0.96–1.01) | 0.389 |
|  |  |  |  |  |  |  |
| **Biliary etiology, prior antibiotics, early infection** | **Rectal** | Detected (>0%) | 25 | 9 | 1.27 (0.48–3.37) | 0.637 |
|  |  | ≥1% | 15 | 6 | 1.77 (0.66–4.75) | 0.257 |
|  |  | ≥2.5% | 14 | 6 | 2.64 (0.95–7.35) | 0.064 |
|  |  | ≥5% | 11 | 6 | 4.26 (1.37–13.26) | 0.012 |
|  |  | ≥7.5% | 11 | 6 | 3.38 (1.01–11.38) | 0.049 |
|  |  | ≥10% | 10 | 5 | 1.58 (0.38–6.53) | 0.528 |
|  |  | Continuous (log₁₀)* |  |  | 1.30 (0.82–2.06) | 0.270 |
|  |  | Inverse Simpson† |  |  | 0.98 (0.96–1.01) | 0.224 |
|  | **Salivary** | Detected (>0%) | 9 | 6 | 6.69 (2.19–20.42) | <0.001 |
|  |  | ≥1% | 5 | 3 | 3.64 (0.78–17.04) | 0.101 |
|  |  | ≥2.5% | 4 | 3 | 4.58 (0.97–21.63) | 0.055 |
|  |  | ≥5% | 3 | 2 | 4.83 (1.00–23.23) | 0.049 |
|  |  | ≥7.5% | 3 | 2 | 4.98 (1.03–23.96) | 0.045 |
|  |  | ≥10% | 3 | 2 | 4.98 (1.03–23.96) | 0.045 |
|  |  | Continuous (log₁₀)* | — | — | 2.04 (1.19–3.49) | 0.009 |
|  |  | Inverse Simpson† | — | — | 0.98 (0.96–1.01) | 0.224 |

| **Table S8 Characteristics of patients with infected necrosis** | | | | | | |
| --- | --- | --- | --- | --- | --- | --- |
| **Subject** | **Day IPN** | **Diagnosis IPN** | **Organism(s) first positive pancreas culture** | **Organism(s) first positive blood culture** | **Days first rectal Enterococcus colonisation / before IPN** | **Days first saliva Enterococcus detection / before IPN** |
| 11 | 42 | GC | - | No bacteremia |  | 6 (36) |
| 13 | 84 | GC, PNC | Escherichia coli  Candida glabrata | No bacteremia |  |  |
| 15 | 42 | PNC | Enterococcus faecium  Candida albicans | Enterococcus faecium | 17 (25) | 5 (37) |
| 26 | 26 | GC, PNC | Klebsiella pneumoniae  Escherichia coli  Morganella morganii | Klebsiella pneumoniae, Escherichia coli | 4 (22) |  |
| 36 | 30 | GC, PNC | Klebsiella pneumoniae | No bacteremia |  | 7 (23) |
| 45 | 11 | PNC | Escherichia coli | Escherichia coli | 9 (2) | 9 (2) |
| 51 | 50 | PNC | Streptococcus salivarius group | No bacteremia |  | 4 (46) |
| 52 | 107 | PNC | Haemophilus parainfluenza  Streptococcus salivarius groep  Streptococcus mitis | No bacteremia | 11 (96) |  |
| 112 | 159 | DPC | - | No bacteremia |  |  |
| 169 | 35 | GC | - | No bacteremia | 17 (18) | 26 (9) |
| 175 | 48 | PNC | - | No bacteremia |  |  |
| 179 | 53 | GC | - | No bacteremia |  |  |
| 188 | 32 | PNC | Enterococcus faecium  Candida kefyr | No bacteremia | 9 (23) | 12 (20) |
| 189 | 70 | PNC | Enterococcus faecalis  Escherichia coli | No bacteremia |  |  |
| 216 | 40 | PNC | Proteus mirabilis, Bacteroides fragilis | Proteus mirabilis |  | 17 (23) |
| 218 | 25 | PNC | Streptococcus anginosus  Fusobacterium ssp  Peptrostreptococcus ssp  Prevotella oris | No bacteremia |  |  |
| 222 | 16 | PNC | Enterococcus faecalis  Staphylococcus epidermidis | Staphylococcus hominis |  | 3 (13) |
| 247 | 10 | GC, PNC | Lactobacillus spp  Candida albicans  Candida glabrata | No bacteremia |  |  |
| 249 | 17 | GC, PNC | - | No bacteremia |  |  |
| 266 | 4 |  | - | No bacteremia |  |  |
| IPN, infected (peri)pancreatic necrosis; GC, gas configuration on imaging; PNC, positive necrosis culture, DPC, drained (peri)pancreatic collections | | | | | | |

| **Table S9 Antibiotic administration** | | | | |
| --- | --- | --- | --- | --- |
|  | | | **Sterile necrosis**  **n=37** | **Infected necrosis n=20** |
| Antibiotics during admission | | | 13 (36.1%) | 20 (100%) |
| Antibiotics before diagnosis necrosis, total (%) | | | 6 (16.7%) | 10 (50.0%) |
| Antibiotics before infected necrosis, total (%) | | | - | 19 (95.0%) |
| Early antibiotics (first week of admission) | | | 6 (16.7%) | 9 (45.0%) |
|  | For documented infection | | 4 (66.7%) | 5 (55.6%) |
|  |  | Bacteremia, total (%) | 2 (33.3%) | - |
|  |  | Pneumonia, total (%) | 2 (33.3%) | 4 (44.4%) |
|  |  | Cholangitis, total (%) | - | 1 (11.1%) |
|  | Without documented infection | | 2 (33.3%) | 4 (44.4%) |
|  |  | Suspected infected necrosis, total (%) | - | 2 (22.2%) |
|  |  | Sterile necrosis, total (%) | - | 2 (22.2%) |
|  |  | Unclear indication, total (%) | 2 (33.3%) | - |
|  | Type antibiotics | | | |
|  |  | Amoxicillin/Clavulanic Acid, total (%) | 1 (16.7%) | 2 (22.2%) |
|  |  | Amoxicillin, total (%) | - | 1 (11.1%) |
|  |  | Cefotaxime, total (%) | 1 (16.7%) | - |
|  |  | Ceftriaxone, total (%) | 3 (50.0%) | 4 (44.4%) |
|  |  | Cefuroxime, total (%) | 2 (33.3%) | 4 (44.4%) |
|  |  | Gentamicin, total (%) | - | 2 (22.2%) |
|  |  | Metronidazole, total (%) | - | 2 (22.2%) |
|  |  | Meropenem, total (%) | - | 2 (22.2%) |

| **Table S10 Linear mixed-effects model ANOVA for microbiome alpha diversity: early antibiotics vs no early antibiotics** | | | | |
| --- | --- | --- | --- | --- |
| **material** | **effect** | **chisq** | **df** | **p_value** |
| rectal_swab | group | 1.413 | 1 | 0.2346 |
| rectal_swab | day | 14.066 | 1 | 0.0002 |
| rectal_swab | group:day | 0.056 | 1 | 0.8137 |
| saliva | group | 10.420 | 1 | 0.0012 |
| saliva | day | 9.553 | 1 | 0.0020 |
| saliva | group:day | 0.187 | 1 | 0.6654 |

| **Table S11 Linear mixed-effects model model coefficients for microbiome alpha diversity: early antibiotics vs no early antibiotics** | | | | | | | |
| --- | --- | --- | --- | --- | --- | --- | --- |
| **material** | **effect** | **term** | **estimate** | **std.error** | **statistic** | **df** | **p.value** |
| rectal_swab | fixed | (Intercept) | 45.085 | 3.815 | 11.819 | 133.764 | <0.001 |
| rectal_swab | fixed | groupyes | -4.341 | 6.510 | -0.667 | 112.633 | 0.506 |
| rectal_swab | fixed | day | -0.942 | 0.360 | -2.615 | 141.455 | 0.010 |
| rectal_swab | fixed | groupyes:day | -0.126 | 0.535 | -0.236 | 147.531 | 0.814 |
| saliva | fixed | (Intercept) | 69.448 | 6.344 | 10.946 | 136.825 | <0.001 |
| saliva | fixed | groupyes | -21.893 | 10.769 | -2.033 | 123.099 | 0.044 |
| saliva | fixed | day | -1.179 | 0.603 | -1.955 | 141.778 | 0.053 |
| saliva | fixed | groupyes:day | -0.381 | 0.880 | -0.432 | 147.055 | 0.666 |

| **Table S12 Linear mixed-effects model results: early antibiotics versus no early antibiotics on microbiome PCoA trajectories** | | | | | | | |
| --- | --- | --- | --- | --- | --- | --- | --- |
| **Material** | **PCoA_Axis** | **Variance_Explained** | **Term** | **Estimate** | **Std_Error** | **t_value** | **P_value** |
| rectal swab | PCo1 | 26.365 | (Intercept) | -0.196 | 0.040 | -4.954 | <0.001 |
| rectal swab | PCo1 | 26.365 | Early antibiotics | 0.108 | 0.067 | 1.604 | 0.111 |
| rectal swab | PCo1 | 26.365 | Day | 0.018 | 0.004 | 4.941 | <0.001 |
| rectal swab | PCo1 | 26.365 | Early antibiotics:Day | -0.003 | 0.006 | -0.496 | 0.621 |
| rectal swab | PCo2 | 12.837 | (Intercept) | 0.106 | 0.029 | 3.707 | 0.000 |
| rectal swab | PCo2 | 12.837 | Early antibiotics | -0.045 | 0.049 | -0.928 | 0.355 |
| rectal swab | PCo2 | 12.837 | Day | -0.009 | 0.003 | -3.468 | 0.001 |
| rectal swab | PCo2 | 12.837 | Early antibiotics:Day | -0.002 | 0.004 | -0.408 | 0.684 |
| rectal swab | PCo3 | 6.776 | (Intercept) | -0.027 | 0.023 | -1.193 | 0.236 |
| rectal swab | PCo3 | 6.776 | Early antibiotics | -0.028 | 0.040 | -0.705 | 0.482 |
| rectal swab | PCo3 | 6.776 | Day | 0.003 | 0.002 | 1.450 | 0.149 |
| rectal swab | PCo3 | 6.776 | Early antibiotics:Day | 0.002 | 0.003 | 0.642 | 0.522 |
| rectal swab | PCo4 | 6.328 | (Intercept) | -0.023 | 0.020 | -1.169 | 0.244 |
| rectal swab | PCo4 | 6.328 | Early antibiotics | 0.005 | 0.034 | 0.142 | 0.887 |
| rectal swab | PCo4 | 6.328 | Day | 0.007 | 0.002 | 3.831 | <0.001 |
| rectal swab | PCo4 | 6.328 | Early antibiotics:Day | -0.011 | 0.003 | -3.957 | <0.001 |
| saliva | PCo1 | 25.290 | (Intercept) | -0.132 | 0.029 | -4.492 | <0.001 |
| saliva | PCo1 | 25.290 | Early antibiotics | 0.008 | 0.049 | 0.158 | 0.875 |
| saliva | PCo1 | 25.290 | Day | 0.009 | 0.003 | 3.054 | 0.003 |
| saliva | PCo1 | 25.290 | Early antibiotics:Day | 0.014 | 0.004 | 3.295 | 0.001 |
| saliva | PCo2 | 16.532 | (Intercept) | 0.007 | 0.029 | 0.227 | 0.821 |
| saliva | PCo2 | 16.532 | Early antibiotics | 0.062 | 0.050 | 1.258 | 0.211 |
| saliva | PCo2 | 16.532 | Day | 0.003 | 0.003 | 1.198 | 0.233 |
| saliva | PCo2 | 16.532 | Early antibiotics:Day | -0.012 | 0.004 | -3.074 | 0.003 |
| saliva | PCo3 | 9.814 | (Intercept) | -0.066 | 0.022 | -2.931 | 0.004 |
| saliva | PCo3 | 9.814 | Early antibiotics | 0.059 | 0.039 | 1.518 | 0.132 |
| saliva | PCo3 | 9.814 | Day | 0.008 | 0.002 | 4.061 | <0.001 |
| saliva | PCo3 | 9.814 | Early antibiotics:Day | -0.009 | 0.003 | -2.917 | 0.004 |
| saliva | PCo4 | 8.208 | (Intercept) | 0.024 | 0.021 | 1.138 | 0.257 |
| saliva | PCo4 | 8.208 | Early antibiotics | -0.060 | 0.036 | -1.696 | 0.093 |
| saliva | PCo4 | 8.208 | Day | -0.003 | 0.002 | -1.586 | 0.115 |
| saliva | PCo4 | 8.208 | Early antibiotics:Day | 0.008 | 0.003 | 2.902 | 0.004 |

| **Table S13 Linear mixed-effects model ANOVA: early antibiotics versus no early antibiotics on butyrate-producer relative abundance** | | | |
| --- | --- | --- | --- |
| **effect** | **chisq** | **df** | **p_value** |
| group | 3.735 | 1 | 0.053 |
| day | 0.873 | 1 | 0.350 |
| group:day | 2.128 | 1 | 0.145 |

| **Table S14 Linear mixed-effects model coefficients: early antibiotics versus no early antibiotics on butyrate-producer relative abundance** | | | | | | |
| --- | --- | --- | --- | --- | --- | --- |
| **effect** | **term** | **estimate** | **std.error** | **statistic** | **df** | **p.value** |
| fixed | (Intercept) | 0.115 | 0.015 | 7.626 | 138.235 | <0.001 |
| fixed | groupyes | -0.007 | 0.026 | -0.273 | 120.655 | 0.786 |
| fixed | day | 0.000 | 0.001 | 0.289 | 139.465 | 0.773 |
| fixed | groupyes:day | -0.003 | 0.002 | -1.459 | 146.002 | 0.147 |

**References**

1. Costea PI, Zeller G, Sunagawa S, et al. Towards standards for human fecal sample processing in metagenomic studies. *Nat Biotechnol* 2017; **35**(11): 1069-76.

2. van den Berg FF, van Dalen D, Hyoju SK, et al. Western-type diet influences mortality from necrotising pancreatitis and demonstrates a central role for butyrate. *Gut* 2021; **70**(5): 915-27.

3. Kozich JJ, Westcott SL, Baxter NT, Highlander SK, Schloss PD. Development of a dual-index sequencing strategy and curation pipeline for analyzing amplicon sequence data on the MiSeq Illumina sequencing platform. *Appl Environ Microbiol* 2013; **79**(17): 5112-20.

4. Edgar RC. Search and clustering orders of magnitude faster than BLAST. *Bioinformatics* 2010; **26**(19): 2460-1.

5. Edgar R. UNOISE2: improved error-correction for Illumina 16S and ITS amplicon sequencing. bioRxiv; 2016.

6. Wang Q, Garrity GM, Tiedje JM, Cole JR. Naive Bayesian classifier for rapid assignment of rRNA sequences into the new bacterial taxonomy. *Appl Environ Microbiol* 2007; **73**(16): 5261-7.

7. Quast C, Pruesse E, Yilmaz P, et al. The SILVA ribosomal RNA gene database project: improved data processing and web-based tools. *Nucleic Acids Res* 2013; **41**(Database issue): D590-6.

8. Reitmeier S, Hitch TCA, Treichel N, et al. Handling of spurious sequences affects the outcome of high-throughput 16S rRNA gene amplicon profiling. *ISME Communications* 2021; **1**(1): 31.

9. Lin H, Peddada SD. Analysis of compositions of microbiomes with bias correction. *Nature Communications* 2020; **11**(1): 3514.

10. Mallick H, Rahnavard A, McIver LJ, et al. Multivariable association discovery in population-scale meta-omics studies. *PLoS Comput Biol* 2021; **17**(11): e1009442.

11. Vujkovic-Cvijin I, Sklar J, Jiang L, Natarajan L, Knight R, Belkaid Y. Host variables confound gut microbiota studies of human disease. *Nature* 2020; **587**(7834): 448-54.

12. Blanche P, Dartigues JF, Jacqmin-Gadda H. Estimating and comparing time-dependent areas under receiver operating characteristic curves for censored event times with competing risks. *Stat Med* 2013; **32**(30): 5381-97.
